# Structural and functional characterization of VapBC52 toxin–antitoxin system from *Mycobacterium tuberculosis*

**DOI:** 10.1093/nar/gkag611

**Published:** 2026-06-17

**Authors:** Manisha Singh, Charandeep Singh, Akshay V Nair, Imran Ahmad, Arun Sharma, Munmun Bhasin, Vikas Jain, Ramandeep Singh, Krishan Gopal Thakur

**Affiliations:** Centre for Tuberculosis Research, Tuberculosis Research Laboratory, BRIC-Translational Health Science and Technology Institute, Faridabad–Gurugram Expressway, Faridabad, Haryana 121001, India; Structural Biology Laboratory, CSIR-Institute of Microbial Technology, Sector 39A, Chandigarh 160036, India; Academy of Scientific and Innovative Research (AcSIR), Ghaziabad 201002, India; Microbiology and Molecular Biology Laboratory, Department of Biological Sciences, Indian Institute of Science Education and Research (IISER) Bhopal, Madhya Pradesh 462066, India; Centre for Tuberculosis Research, Tuberculosis Research Laboratory, BRIC-Translational Health Science and Technology Institute, Faridabad–Gurugram Expressway, Faridabad, Haryana 121001, India; Manipal Academy of Higher Education, Manipal, Karnataka 576104, India; Centre for Tuberculosis Research, Tuberculosis Research Laboratory, BRIC-Translational Health Science and Technology Institute, Faridabad–Gurugram Expressway, Faridabad, Haryana 121001, India; Molecular Biophysics Unit, Indian Institute of Science, Bangalore 560012, India; Laboratory of Antimicrobial Innovation, School of Interwoven Arts and Sciences, Krea University, Sri City, Andhra Pradesh 517646, India; Centre for Tuberculosis Research, Tuberculosis Research Laboratory, BRIC-Translational Health Science and Technology Institute, Faridabad–Gurugram Expressway, Faridabad, Haryana 121001, India; Structural Biology Laboratory, CSIR-Institute of Microbial Technology, Sector 39A, Chandigarh 160036, India; Academy of Scientific and Innovative Research (AcSIR), Ghaziabad 201002, India; Department of Biotechnology, National Institute of Pharmaceutical Education & Research (NIPER), Sector 67, S.A.S, Nagar, Punjab 160062, India

## Abstract

*Mycobacterium tuberculosis* (*Mtb*) encodes a huge repertoire of toxin–antitoxin (TA) systems, many of which remain uncharacterized. Here, we report the crystal structures of the VapC52 toxin and VapBC52 TA complex at a resolution of 2.6 and 3.2 Å, respectively. We show that VapC52 adopts a unique open dimeric conformation and inhibits mycobacterial growth by cleaving tRNA at the variable or anticodon loop region. Structure reveals that VapB52 adopts a distinct structural architecture and binds VapC52 with a 1:2 stoichiometry, respectively. Interestingly, binding of ssDNA activates VapB52 peptidase domain, resulting in auto-cleavage of VapB52 N-terminal domain which is critical for VapBC complex formation and neutralization. In addition to VapB52, co-expression of several other non-cognate VapB antitoxins abrogates the growth inhibition associated with VapC52 overexpression in *Mycobacterium smegmatis (Msm*) suggesting crosstalk among VapBC TA systems. Further, we demonstrate that the *vapBC52* locus is dispensable for *in vitro* growth but essential for *Mtb* intracellular growth in macrophages and guinea pigs. Notably, VapC52 also cleaves mycobacteriophage D29 encoded tRNAs and confers resistance to phage infection in *Msm*. Taken together, we show that VapBC52 adopts a unique structural architecture, plays role in pathogenesis, and is possibly involved in mycobacterial antiphage defense mechanisms.

## Graphical Abstract

**Graphical Abstract:**
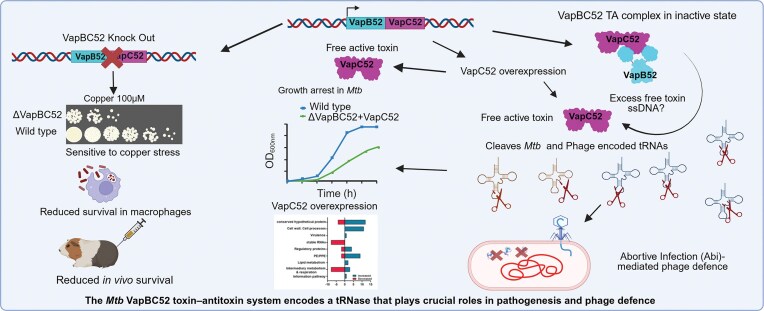

## Introduction

Toxin–antitoxin (TA) systems are ubiquitous gene loci in bacterial genomes that encode a stable toxin and an antitoxin [1]. TA systems are classified into eight types based on mechanistic diversity [2]. Among these, Type II TA systems, in which both toxin and antitoxin are proteins that physically interact under normal conditions are extensively studied [3]. The antitoxin auto-regulates TA system transcription in addition to neutralizing the activity of its corresponding toxin [1]. The toxin generally interferes with essential cellular functions, such as translation, DNA replication, and cell wall synthesis, resulting in reversible growth arrest [4, 5]. Although TA systems impose a significant metabolic burden on the cell, their widespread presence in pathogens undergoing genome reduction suggests broader physiological significance [6, 7]. The majority of the annotated TA systems in the genome of *Mycobacterium tuberculosis* (*Mtb*) belong to the VapBC family [7, 8]. VapC toxins are characterized by the presence of a conserved PIN (PilT N-terminus) domain and function as Mg²⁺/Mn²^+^-dependent endoribonucleases [9]. VapC toxins cleave the anticodon loops of specific tRNAs or the sarcin–ricin loop of 23S rRNA [10–16].

There have been studies describing structural details or functions of TA systems in mycobacterial physiology, stress adaptation, or pathogenesis [12, 17–30]. These studies indicate that whereas subset of TA systems is essential for host adaptation and pathogenesis, others can have context-specific or redundant functions. These studies also demonstrate diversity in structures and mechanisms of toxin neutralization observed in these TA systems. Besides bacterial stress adaptation, or virulence, emerging evidence highlights that a subset of TA systems is involved in antiphage defense [31–40]. Despite being an intracellular pathogen, recent studies suggest that *Mtb* can survive in a variety of environmental conditions, such as soil, water, etc., where it might encounter potential phage infections [41]. Despite the relative abundance of TA systems in *Mtb*, their potential contribution to phage resistance has not yet been investigated. This gap is particularly intriguing given an unusually large number of chromosomal TA *loci* in *Mtb* and the fundamental need to understand bacterial mechanisms that may counteract phage infection, especially in light of the growing clinical interest in phage therapy and survival during transmission [42, 43].

Tandon *et al*. have established that Rv2514c–Rv2515c encode for a functional TA system [7]. Here, we report detailed structural, biochemical, and functional characterization of Rv2514c–Rv2515c TA system, henceforth referred as VapC52 and VapB52, respectively. We have determined the crystal structures of VapC52 and VapBC52 TA complex, revealing a distinct mode of toxin dimerization and mechanism of toxin neutralization. We also show that VapB52 is having unique domain organization and undergoes self-cleavage in the presence of ssDNA. Using co-expression studies, the growth inhibition associated with VapC52 overexpression in *M. smegmatis (Msm)* was neutralized by co-expression of VapB52 and several other non-cognate VapB antitoxins. We show that deletion of *vapBC52* significantly impaired the growth of *Mtb* in guinea pigs. Further, overexpression of VapC52 in *Msm* enhanced resistance to mycobacteriophage infection, suggesting a function in antiphage defense. Additionally, we demonstrate that VapC52 cleaves both mycobacterial and phage-derived tRNAs. Overall, we have demonstrated that VapBC52 is a structurally distinct member of the VapBC family that contributes to disease pathogenesis and possibly has a role in antiphage defense.

## Material and methods

### Bacterial strains and culture conditions

*Escherichia coli* strains used in this study [XL-1 blue, Top10, Rosetta (DE3), and HB-101] were cultured in LB (Luria–Bertani) broth or LB–Agar at 37 °C, 200 rpm. *Msm* mc^2^155 and *Mtb* H37Rv strain were cultured in Middlebrook (MB) 7H9 medium supplemented with 0.2% glycerol, 1× albumin–dextrose–saline (ADS), 0.05% Tween-80 at 37 °C, 150 rpm. Mycobacterial enumeration and streaking of strains were performed on MB7H11 agar supplemented with 1× oleic acid–ADS (OADS) at 37 °C for 2–3 days (*Msm*) or 3–4 weeks (*Mtb*). Wherever required, the growth medium was supplemented with appropriate antibiotics using the following concentrations: hygromycin (50 μg/mL for mycobacteria and 150 μg/mL for *E. coli*), kanamycin (25 μg/mL for mycobacteria and *E. coli*), ampicillin (50 μg/mL for *E. coli*), tetracycline (10 μg/mL for *E. coli*), and apramycin (50 μg/mL for *E. coli*).

### Cloning and protein expression

The DNA fragments coding for either *vapB52* or *vapC52*, were cloned in pET28b between NdeI and XhoI restriction sites. VapC52 harboring mutations in PIN domain (VapC52^D6A^, VapC52^E48A^, VapC52^D99A^ VapC52^E117A^) or Zn^2+^ binding motif (VapB52^H216A,H220A^) were created using PCR-based site-directed mutagenesis as per standard protocols. For VapB52 purification, both full-length and 23-residue amino-terminus truncated construct (VapB52^V24-V415^, named VapB52^Δ23^) were designed to remove disordered region based on PSIpred analysis [44] and were used for protein purification. For co-expression and co-purification of VapB^Δ23^C52, the pETDuet-N vector was used [11]. Firstly, *vapB52*^Δ23^ was cloned into the NdeI and XhoI sites of MCS-2 followed by cloning of *vapC52* into MCS-1 between NheI and HindIII sites. Similarly, for co-expression and co-purification studies, *vapB52*^ΔNTD^ (T93-V415) and *vapB52*^ΔCTD^ (V24-G357) were cloned in MCS-2 followed by cloning of *vapC52^D6A^* in MCS-1. Rosetta (DE3) (Novagen, Germany) cells were used for the expression of VapC52^D6A^, VapB52^Δ23^, and VapB^Δ23^C52. Transformed Rosetta (DE3) cells were plated on LB agar plates with specific antibiotics and incubated at 37 °C overnight. The primary culture (10 mL of LB) was inoculated with a single colony from the transformed plate and incubated overnight with constant shaking of 200 rpm at 37 °C. For secondary culture, 1% of the primary culture was inoculated, and cells were induced by adding 0.3 mM Isopropyl β-D-1-thiogalactopyranoside (IPTG) at OD_600nm_ of 0.4–0.6 and further incubated at 16 °C for 18 h at a constant shaking of 170 rpm. The induced cultures were harvested by centrifugation at 6000 × *g* for 15 min at 4 °C, and the pellets were re-suspended in lysis buffer [20 mM 4-(2-hydroxyethyl)-1-piperazineethanesulfonic acid (HEPES) pH 7.5, 150 mM NaCl, and a cocktail of EDTA-free protease inhibitors] followed by sonication. The supernatant was collected after centrifugation at 18 000 × *g* for 45 min. The 6x His-tagged proteins were further purified using Ni-NTA affinity chromatography as per the manufacturer’s recommendations. The purity of fractions was assessed on SDS–PAGE. The desired elution fractions were pooled and concentrated using ultrafiltration centrifugal devices (Amicon, Merck Millipore, USA). The purified proteins were further purified by gel filtration chromatography using Superdex^™^ 200 Increase 10/300 GL (GE Healthcare, USA) column in a buffer containing 20 mM HEPES, pH 8.0 and 150 mM NaCl. The identities of the protein samples were verified by mass spectrometry, and the purity of the samples was examined using SDS–PAGE.

### Crystallization and data processing of VapC52^D6A^ and VapB^Δ23^C52

The initial crystallization trials for VapC52^D6A^ and VapB^Δ23^C52 complex were performed using commercially available screens from Hampton Research, USA and Molecular Dimensions, UK. Sitting-drop vapour diffusion crystallization trials were set in a high-throughput 96-well crystallization tray, manually mixing equal volumes of reservoir buffer and protein (0.7 μL each). The plates were incubated at 20 °C and imaged at regular intervals using automatic prescheduled imaging in Rock Imager 1000 (Formulatrix, USA). The crystals for VapC52^D6A^ (5.5 mg/mL) appeared in 1.5 M ammonium sulfate, 0.1 M tris, pH 8.5 and 12% v/v glycerol after 7 days of incubation. X-ray diffraction data for VapC52^D6A^ toxin crystals were collected at PX-BL21 beamline, RRCAT, Indore, India at 100 K temperature. The VapC52^D6A^ crystal X-ray data was collected from a single crystal at a wavelength of 0.9789 Å and processed to a resolution of 2.6 Å. X-ray diffraction data for the VapC52^D6A^ toxin were processed using XDS suite and scaled using Aimless [45, 46]. The Phaser module in the CCP4 software suite was used to solve the structure using the molecular replacement method [45, 47]. An AlphaFold 3 [45, 48] predicted model was used as a search template. Iterative model building using COOT [49] and structure refinement using Refmac5 [45] resulted in *R*_work_/*R*_free_ of 0.171/0.229. The structure validation was performed using MolProbity [50]. The models had excellent geometry, and the Ramachandran Plot showed no outliers.

The VapB^Δ23^C52 complex (15 mg/mL) crystallized in a condition containing 0.8 M potassium sodium tartrate tetrahydrate, 0.1 M sodium HEPES pH 7.5, after two weeks of incubation. The X-ray diffraction data for VapB^Δ23^C52 complex crystals were collected at Diamond Light Source UK, Beamline I04 at 100 K temperature. The final data were collected at a wavelength of 0.9537 Å and processed to 3.2 Å resolution using iMOSFLM and scaled using SCALA [45, 51, 52]. The VapB^Δ23^C52 complex structure was solved by the molecular replacement method in Phaser using the crystal structure of VapC52 and the AlphaFold 3 predicted structure of VapB^Δ23^52 as search templates [45, 48]. Iterative manual model building was done using COOT and structure was refined using Phenix.Refine [49, 53]. The structure was refined to final *R*_work_/*R*_free_ of 0.254/0.280. The data collection, processing and refinement parameters are provided in the Supplementary Table S1. PyMOL and COOT were used to visualize and analyse crystal structures [49]. Structural homology searches for both VapC52 and VapB^Δ23^C52 were performed using the PDBeFold server (http://ebi.ac.uk/msd-srv/ssm/cgi-bin/ssmserver/). The oligomeric states and interface contacts in VapC52 and VapB^Δ23^C52 were identified using the PDBePISA online server at http://www.ebi.ac.uk/pdbe/pisa/.

### BIOSAXS data collection

Size-exclusion chromatography coupled with small-angle X-ray scattering (SEC-SAXS) data for VapC52^D6A^ and VapB^Δ23^C52 were collected at BM29, European Synchrotron Radiation Facility (ESRF), Grenoble, France. In-line SEC-SAXS experiments were performed using the Advance BioSEC 300 7.8/300 (Agilent, USA) column. The data collected were processed using ATSAS 3.0 software suite to obtain the radius of gyration (*R*_g_), the pair distribution function [*P*(*r*)], and the dimensionless Kratky plot [54]. The molecular weight from BIO-SAXS data was calculated by dividing the Porod volume by a conversion factor of 1.3. GASBOR was used to generate *ab initio* dummy atom models for the protein samples, which were fitted in the solved crystal structures using SUPALM [54]. P2 and P1 symmetry were used to generate dummy atom *ab initio* models for VapC52^D6A^ and VapB^Δ23^C52, respectively. The data were plotted using OriginPro 2016 (OriginLab Corporation) software package.

### Isothermal titration calorimetry

To determine *K*_d_, stoichiometry and thermodynamics of the VapB^Δ23^52 and VapC52 complex, ITC experiments were performed at 25 °C using MicroCal Auto-iTC200 (Malvern MicroCal, LLC). Both VapB52^Δ23^ and VapC52^D6A^ were purified by size-exclusion chromatography in buffer containing 20 mM HEPES, pH 7.5, 250 mM NaCl, and 10% glycerol. For ITC experiments, VapB52^Δ23^ (25 μM, sample cell) was titrated with VapC52^D6A^ (250 μM, syringe) at a constant stirring rate of 750 rpm. A total of 30 injections of 1 μL (0.4 μL for the first injection) were administered at intervals of 120 s, with a reference power of 10 µCal/s and a filter period of 5 s. The data obtained was fitted using a one-site binding model using the OriginPro 2016 (OriginLab Corporation, USA) software package.

### Pull-down assay

For pull-down assays, co-expression followed by a co-purification strategy was used. The *vapC52^D6A^* was cloned in MCS-1 and deletion variants of *vapB52^Δ23^* were cloned in MCS-2 of the pET-Duet-N vector, resulting in pETDuet-N-VapB^Δ23^,^ΔNTD^C52^D6A^, pETDuet-N-VapB ^Δ23,ΔCTD^C52^D6A^, and pETDuet-N-VapB52 ^Δ23,H216A,H220A^C52^D6A^ constructs. Protein expression and purification were performed as described above under the section ‘Cloning and Protein expression’. The elution fractions were analyzed using SDS–PAGE.

### VapB52 self-cleavage assay

For evaluating self-cleavage activity in VapB52, 15 µM of each of purified VapB52^Δ23^ and VapB52^Δ23,H216A,H220A^ proteins were incubated with 15 µM of oligo(dT) (24 mer) and random ssDNA (24 mer) in buffer (20 mM HEPES, pH 7.5, 150 mM NaCl). A total of 20 µL of each reaction was set and incubated at room temperature for 30 min. The reaction was stopped by adding protein loading dye (1× final concentration) and heating the sample at 99°C for 5 min. For pull-down assay, a total of 300 µL of mixture, as described above, was incubated with 100 µL of Ni-NTA beads. The beads were washed with 1 mL of wash buffer containing 20 mM HEPES pH 7.5, 250 mM NaCl, and 10 mM imidazole. The bound protein was eluted using wash buffer supplemented with 250 and 500 mM imidazole. The samples were analysed on 15% SDS–PAGE.

### Construction of knockout strains

*Mtb strains* lacking either *vapC52* or *vapBC52* loci were constructed using temperature-sensitive mycobacteriophages as previously described [55]. Briefly, for generating Δ*vapC52* or Δ*vapBC52* strains, approximately 800 base pairs upstream and downstream regions of either *vapC52* or *vapBC52* were PCR amplified and cloned into pYUB854 flanking the hygromycin resistance cassette. The modified cosmids, pYUB854-Δ*vapC52* and pYUB854-Δ*vapBC52*, were *PacI* digested and packaged into pYUB159 using MaxPlax Lambda Packaging extracts. The recombinant phagemid was electroporated into *Msm* to generate high-titre temperature-sensitive mycobacteriophages. To generate Δ*vapC52* or Δ*vapBC52* in *Mtb* H37Rv, mid-log phase cultures (OD_600nm_ ∼ 0.8–1.0) were transduced with temperature-sensitive mycobacteriophages as reported earlier [56]. The generation of mutant strains was verified by Southern blot using locus-specific probes. For qPCR studies, total RNA was isolated from mid-log phase cultures of various strains and subjected DNase I treatment to remove contaminated DNA. Further, cDNA was synthesized using Superscript III reverse transcriptase in accordance with the manufacturer’s recommendations. The synthesized cDNA was subjected to qPCR using gene-specific primers and SYBR green qPCR Master mix as per standard protocols. The transcript levels were normalized to *sigA* levels, a housekeeping gene, and the relative fold change was calculated.

### Growth inhibition assays in *Mtb*

For overexpression studies, *vapC52* ORF was PCR amplified using *Mtb* genomic DNA as a template and cloned into anhydrotetracycline (Atc) inducible integrative vector (pTetR-Int). For growth inhibition assays in *Mtb*, pTetR-int or pTetR-int-*vapC52* was electroporated in Δ*vapBC52*, and transformants were selected on MB7H11 plates supplemented with kanamycin and hygromycin. For overexpressing VapC52, the early-log phase cultures of recombinant strains (OD_600nm_ ∼0.2–0.3) were induced by adding 50 ng/mL Atc, and growth was monitored till stationary phase was attained.

### RNA-seq data processing

For RNA sequencing, early-log phase cultures of Δ*vapBC52* (OD_600nm_ ∼0.2–0.3) harboring either pTetR-int or pTetR-int *vapC52* were induced by adding Atc (50 ng/mL) for 24 h. Induced cultures were harvested at 24 h post-induction and washed twice with 1× PBS. The washed pellet was re-suspended in 1 mL of Trizol (Ambion, Inc), followed by bead-beating with intermittent cooling for cell lysis. Total RNA was extracted from the clarified lysate using the phenol–chloroform, precipitated with isopropanol and purified using the Qiagen RNA isolation kit as per the manufacturer’s instructions. The purified RNA was shipped to Bionivid, India for RNA sequencing. Further DNase I treatment and ribosomal RNA depletion were performed. The cDNA libraries were prepared using the NEBNext^®^ UltraTM II Directional RNA Library Prep Kit for Illumina and sequenced using the Nova Seq 6000 platform. Further analysis of the data obtained from RNA-seq experiments was performed as previously reported [23].

### RNA cleavage assay and tRNA sequencing

For *in vitro* ribonuclease assays, template for *Mtb* tRNAs*-* tRNA^ValU(GAC)^, tRNA^ValT(CAC)^, tRNA^SerV(GGA)^, tRNA^SerX(CGA)^, and phage tRNAs- tRNA^Asn(GTT)^, tRNA^Trp(CCA)^, tRNA^Gln(CTG)^, tRNA^Glu(CTC)^, and tRNA^Tyr(GTA)^ were generated by self-annealing forward (having T7 promoter at the 5′) and reverse primers (Supplementary File S1) [57]. tRNA DNA templates were further PCR amplified using T7 forward primer and tRNA-specific reverse primers. The amplified PCR products were separated on 3% agarose gel and purified using a GeneJET Gel Extraction kit (Thermo Scientific). Further, *in vitro* transcription reaction was performed using T7 RNA polymerase (Takara, Japan) to synthesize tRNAs. For *in vitro* transcription assays, ∼250 ng of purified DNA was used as a template in a final reaction volume of 20 μL as per the manufacturer’s protocol (Takara, Japan). The transcribed products were separated using the 15% urea PAGE and purified using the ZR small-RNA^™^ PAGE recovery kit (Zymo, USA). The tRNA samples were refolded by heating to 95 °C and allowing them to cool to 65 °C in a thermomixer (Eppendorf, Germany). The ribonuclease assays, using transcribed tRNAs, were performed in cleavage buffer (10 mM HEPES pH 8.0, 15 mM KCl, 1 mM dithiothreitol (DTT) , and 20 μM MnCl_2_) containing 15 μM of protein at 37 °C for 45 min. The reaction was stopped by the addition of formamide RNA loading dye and heating the sample at 70 °C for 5 min. The samples were resolved on 15% urea–PAGE gel and visualized by ethidium bromide (EtBr) staining.

For identifying the tRNA cleavage site, the RNA cleaved products were recovered from the 15% urea PAGE using ZR small-RNA^™^ PAGE recovery kit (Zymo, USA). The recovered RNA products were polyadenylated at 3′ end using 0.5 unit of Poly(A) Polymerase (Takara) in 1× buffer [50 mM Tris–HCl, pH 7.9, 10 mM MgCl_2_, 2.5 mM MnCl_2_, 250 mM NaCl, 1 mM DTT, 0.05% BSA, and 1 mM ATP) and incubating sample at 37 °C for 3 h. Next, 3′ polyadenylated tRNA fragments were used to synthesize the complementary DNA (cDNA) using oligo (dT) primer provided in the cDNA synthesis kit (Invitrogen, USA). cDNA was further amplified by Phusion High-Fidelity DNA Polymerase (Thermo Scientific, USA) using tRNA-specific forward primer with 15 annealing bases at the 5′ end of tRNA and a large overhang of 77 bases (including T7 promoter sequence at the 5′ end of the primer) and oligo(dT) reverse primer. The PCR products were gel-purified and sequenced by Sanger sequencing using the T7 promoter primer.

### Growth inhibition and crosstalk studies

The recombinant plasmid, pTetR-int-*vapC52* was electroporated into *Msm*, and transformants were selected on MB7H11 agar plates supplemented with kanamycin. Further expression of VapC52 was induced by the addition of 50 ng/mL anhydrotetracycline (Atc) in early log phase cultures (OD_600nm_ ∼ 0.2–0.3) of *Msm*. For growth restoration assays, *vapB52* or type II non-cognate antitoxins belonging to either VapB, MazE, RelB, or ParD subfamilies were PCR-amplified and cloned into an acetamide inducible episomal vector, pLam12. The resulting plasmids were electroporated into *Msm* harboring an integrative copy of *vapC52* and selected on MB7H11 plates supplemented with kanamycin and apramycin. The overexpression of toxin and antitoxins was induced by the addition of 50 ng/mL Atc and 0.2% acetamide, respectively, and growth patterns of various recombinant strains were determined by measuring either OD_600nm_ or CFU enumeration. For CFU enumeration, 10-fold serial dilutions were prepared and plated on MB7H11 agar plates for 2–3 days.

### Stress experiments and copper susceptibility experiments

To investigate the role of VapBC52 in *Mtb* physiology and stress adaptation, early log phase cultures (OD_600nm_ ∼ 0.2–0.3) of various strains were subjected to different stress conditions such as oxidative (5 mM H_2_O_2_ in MB7H9 medium for 72 h), nitrosative (MB7H9 medium pH-5.2 with 5 mM NaNO_2_ for 72 h), starvation (1× tris buffered saline with 0.05% Tween-80 for 7 or 14 days), as previously reported [58]. For bacterial enumeration, at designated time points, 10.0-fold serial dilutions were prepared and plated on MB7H11 agar plates at 37 °C for 3–4 weeks. For copper tolerance assays, various strains were grown till mid-log phase (OD_600nm_ ∼ 0.8–1.0), washed twice with 1× PBS and spotted on MB7H11 in the absence or presence of 100 µM copper sulphate (CuSO_4_) or 100 µM CuSO_4_^+^ 100 µM bathocuproinedisulfonic acid (BCS) at 37 °C for 3–4 weeks.

### Macrophage infection experiments

For the intracellular survival assays, THP-1 monocytes were seeded at a density of 2 × 10^5^ cells per well and differentiated into macrophages with the addition of 20 ng/mL phorbol 12-myristate 13-acetate for 48 h. The differentiated macrophages were washed twice with 1× PBS. After 24 h, the cells were infected with a single cell suspension prepared from mid-log phase cultures of various *Mtb strains* at a multiplicity of infection (MOI) of 1:10. After infection for 4 h, the extracellular bacteria were removed by overlaying macrophages with RPMI medium containing amikacin (200 µg/mL) for 2 h. Subsequently, the cells were washed twice with 1× PBS and overlaid with RPMI medium. For CFU enumeration, at designated time points, infected macrophages were lysed using 1× PBS containing 0.1% triton-100 (1× PBST), and 10.0-fold serial dilutions were prepared and plated on MB7H11 plates at 37 °C for 3–4 weeks.

### Animal experiments

The animal experiments were performed as per the guidelines of the committee for the purpose of control and supervision of experiments on animals (CCSEA). The animal ethical committee of the BRIC-Translational Health Science and Technology Institute (THSTI) approved the animal experiments (THSTI/IAEC/106). For aerosol infections, various strains were cultured till mid-log phase (OD_600nm_ 0.8–1.0), washed twice with 1× PBS and single-cell suspensions were prepared. The inoculum was diluted subsequently to 1 × 10^8^ CFU in 10 mL saline. For animal experiments, 6- to 8-week-old guinea pigs (Duncan Hartley strain) were infected using Glas-Col aerosol inhalation exposure chamber, which resulted in implantation of 50–100 bacilli in lung tissues. The extent of disease progression and bacillary loads in guinea pigs was determined by CFU enumeration at 4 weeks and 8 weeks post-infection. For CFU enumeration, lungs and spleens were homogenized in 2 mL normal saline, and 10.0-fold serial dilutions were prepared and plated on MB7H11 at 37 °C for 3–4 weeks.

### Phage infection experiments

The primary cultures of *Msm* strains harboring (pTetR-int or pTetR-int-*vapC52, vapC35, vapC31, vapC25, vapC21, mazF3, mazF9*, pTetR-int*-vapC52^D6A^*, pTetR-int*-vapC52 + pLam12 vapB52*) were cultured in MB7H9 medium supplemented with 2% glucose and 0.05% Tween-80 with the appropriate antibiotics at 37 °C with constant shaking till 48 h. The secondary cultures were inoculated at a final OD_600nm_ ∼ 0.1 into MB7H9 supplemented with 2% glucose and 4% OADC and incubated at 37 °C with constant shaking. At OD_600nm_ ∼ 0.4, the expression of antitoxin and toxin was induced with the addition of 0.2% acetamide and 40 ng/mL Atc, respectively, for 2 h. Further, the induced cultures were supplemented with 1 mM CaCl_2_, mixed with D29 mycobacteriophage lysate at an MOI of 1:1, and kept for adsorption at 37 °C without shaking for 20 min. The growth of the cultures was monitored by measuring the OD_600nm_ after every 2 h till 8 h of phage infection. For bacterial viability, 500 µL of the samples were collected at the designated time points, washed twice, and re-suspended in 600 µL of 1× PBST. Further, 180 µL of sample was mixed with 20 µL of Alamar blue reagent (Bio-Rad) and incubated in the dark at 37 °C, 200 rpm. After incubation for 1 h, fluorescence was determined at 495 nm excitation and 524 nm emission wavelengths using a Spectramax M5 plate reader.

### Statistical analysis

GraphPad Prism 8.0.1 software (GraphPad Software) was used for the preparation of graphs and statistical analysis. The *P-*value of < 0.05 was considered to be statistically significant. The statistical test used to analyse the data is mentioned in the respective figure legend.

## Results

### Crystal structure of VapC52 reveals a PIN domain architecture

**Figure 1.**
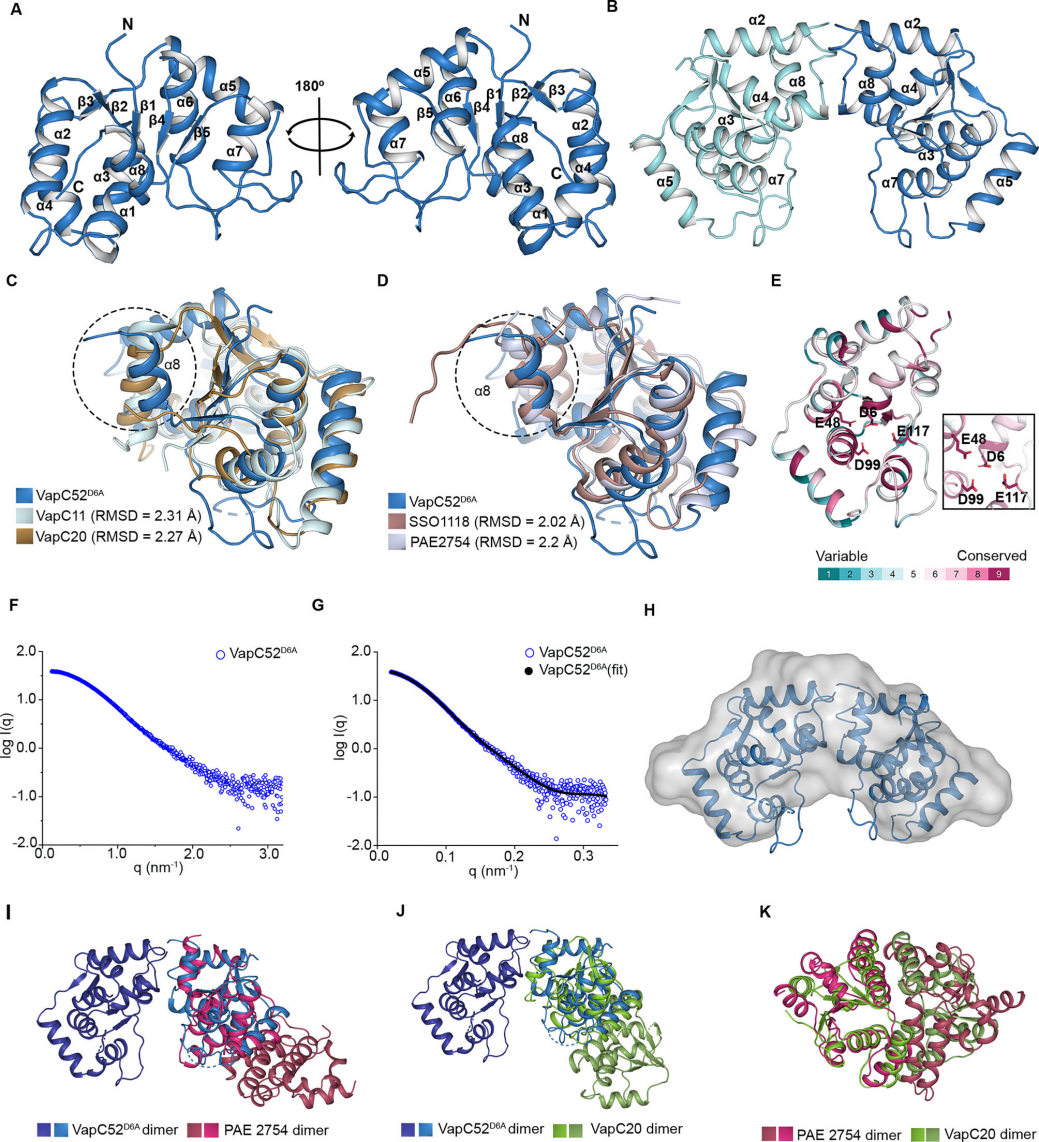
Crystal and solution structure of VapC52^D6A^. (**A**) Crystal structure showing secondary structural elements in VapC52^D6A^. (**B**) Dimeric assembly of VapC52^D6A^ observed in the crystal structure (protomers are shown in cyan and blue).(**C**) Structural superposition of VapC52 with other *Mtb* toxins, VapC11, and VapC20. The dotted circle shows the α8 helix. (**D**) Structural superposition of VapC52 with other PIN domain-containing DUF4411 proteins. The α8 helix in VapC52 is highlighted (dotted circle). (**E**) ConSurf analysis showing conserved and variable regions in VapC52. Highly conserved PIN domain residues are shown in stick representation (left pane and right panels). (**F**) SAXS scattering profile of the VapC52^D6A^. (**G**)The experimental SAXS intensity data (blue circles) and fit of the *ab initio* GASBOR model (black line) for VapC52. (**H**) Fit of VapC52 dimer crystal structure into the SAXS-derived envelope (χ2 = 1.14). (**I** and **J**) Structural superposition of VapC52^D6A^ dimer with PAE2754 (I) and VapC20 dimer (J) showing the distinct open dimeric interface of the former. (**K**) Comparison of VapC20 dimer and PAE2754 dimer, highlighting compact dimeric arrangement of PIN domain proteins.

To elucidate the structure and function of VapC52, an active site mutant variant VapC52^D6A^ was designed based on conserved PIN domain residues, purified, and its crystal structure was determined at a resolution of 2.6 Å (Fig. 1A and B, and Supplementary Fig. S1A and B). VapC52^D6A^ crystallized in the I2_1_3 space group with four molecules in the asymmetric unit (Supplementary Fig. S1C). Electron densities were well resolved for majority of the protein; however, residues E91–R94 (chain A); R90–R94 (chain B); V89–R95, S120–R127 (chain C); and L70–F101, G121–R127 (chain D) were not well resolved most likely as a result of disorder. The crystal structure revealed that VapC52^D6A^ adopts a canonical PIN (PilT N-terminal) domain fold, with an α/β/α sandwich architecture, that consists of five parallel β-strands flanked by three α-helices on one side and five α-helices on the opposite side (Fig. 1A) [59]. Structural homology search using PDBefold server revealed that VapC52^D6A^ shares structural similarity with other *Mtb* VapC toxins such as VapC11 [12], VapC20 [11], VapC43 [60], VapC30 [61], VapC26 [22], and VapC3 [19], as well as other PIN domain containing proteins such as PAE2754 [62] and SSO1118 [63] (Fig. 1C and D, and Supplementary Table S2). Structural comparison revealed variations in the antitoxin-binding interface and the architecture of the active-site pocket, while the overall fold among these proteins remained well conserved [11, 22, 64]. Although VapC52 and other VapCs share a high degree of structural similarity, the C-terminal region (L142-Q149) adopts an α-helical (α8) conformation and this region is absent in other *Mtb* VapC homologs. However, two other PIN-domain proteins, PAE2754 and SSO1118, have similar α-helical conformation at the C-terminal region (Fig. 1C and D). ConSurf and multiple sequence alignment analyses revealed high conservation of the active-site residues, while the remaining regions of the protein are comparatively less conserved (Fig. 1E and Supplementary Fig. S1A).

### VapC52^D6A^ self-associates to form a homodimer with a unique binding interface

The PDBePISA analysis of the VapC52^D6A^ crystal structure revealed a unique and stable dimeric interface formed *via* a two-fold crystallographic symmetry operation. This interface area is approximately 1530 Å² with the active site of each VapC52^D6A^ protomer oriented away from each other (Fig. 1B, and Supplementary Fig. S1D and Supplementary Table S3A). The dimeric interface in VapC52^D6A^ is formed by ɑ8 and the loop connecting ɑ1–ɑ2 and is stabilized by a multitude of interactions such as hydrogen bonds, salt bridges, and non-bonded contacts (Supplementary Fig. S1E and F). Studies have shown that VapC toxins usually form compact homodimers in solution where active sites of both monomers are on the same face [11, 21, 22, 64, 65]. However, an open dimeric conformation was observed in VapC52^D6A^, and this unique conformation could be attributed to the presence of ɑ8 helix (Fig. 1B and Supplementary Fig. S1E).

To further determine the in solution oligomeric state of VapC52^D6A^, we performed SEC–SAXS experiments (Fig. 1F and Supplementary Fig. S2A–E). The molecular weight of ∼38.54 kDa, estimated from the porod volume (50 107 Å^3^), was consistent with the anticipated 38.5 kDa for VapC52^D6A^ homodimer. An *ab initio* dummy atom model fitted well with the experimental SAXS data and also superimposed well on the solved structure of VapC52^D6A^ dimer with a Normalized Spatial Discrepancy (NSD) of 2.34 (Fig. 1H and Supplementary Fig. S2E). Our PDBePISA analysis, BIO-SAXS experiments and comparative structural analysis of VapC52 with other known VapCs and PIN domain proteins confirm that VapC52 self-associates to form a dimer in solution and possesses a unique overall conformation and binding interface (Fig. 1I–K and Supplementary Table S3A).

### VapB52:VapC52 forms a unique 1:2 stoichiometric complex

**Figure 2.**
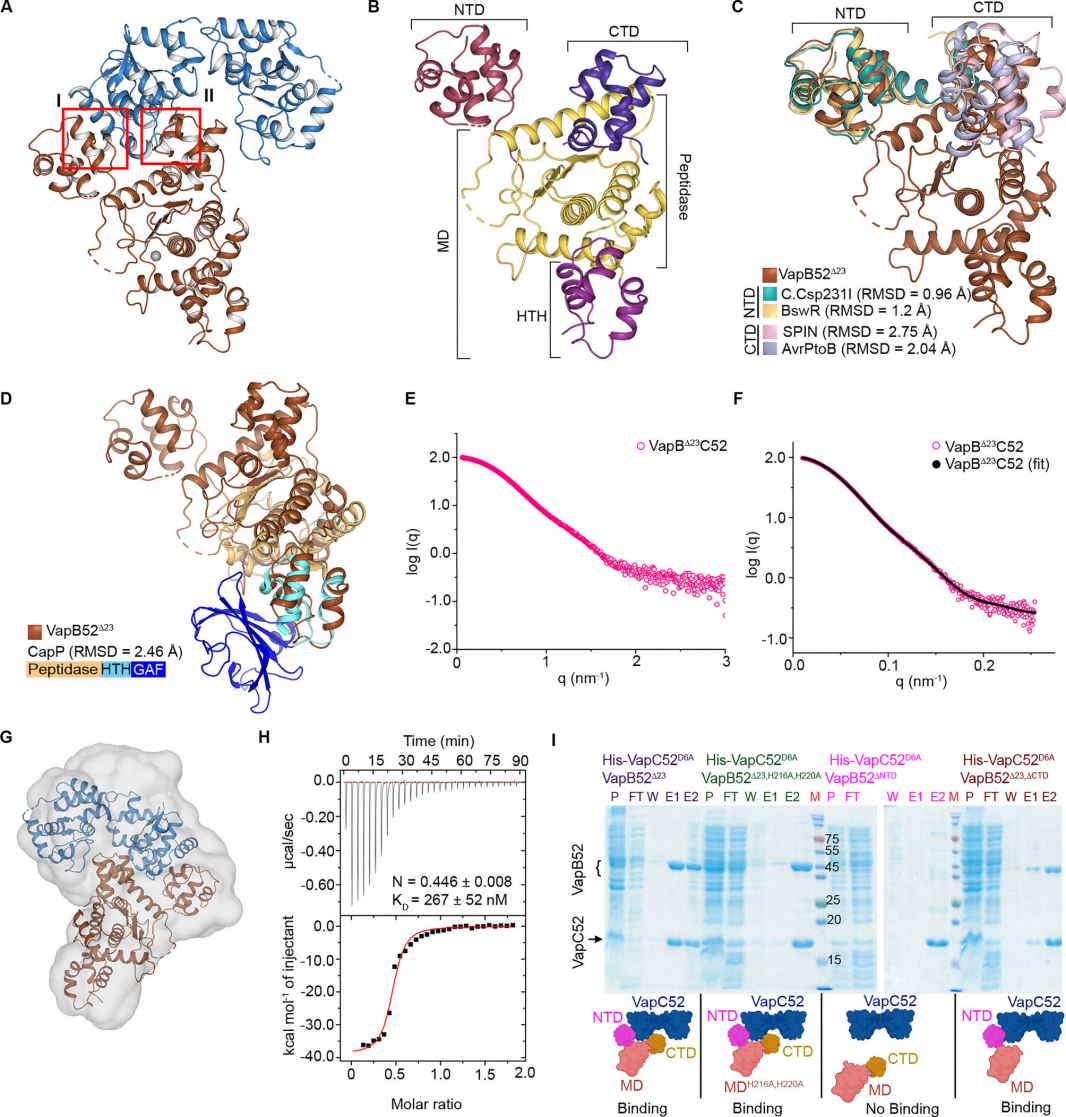
Crystal and solution structures of VapB^Δ23^C52. (**A**) Crystal structure of VapB^Δ23^C52 complex showing two major interacting interfaces, Interface I and Interface II (Supplementary Fig. S3F) shown in red boxes. The Zn^2+^ ion is shown as a grey sphere. (**B**) Domain organization in VapB52^Δ23^ comprising the N-terminal domain (VapB52^NTD^, raspberry), the C-terminal domain (VapB52^CTD^, deep blue), and the middle domain (VapB52^MD^) containing a peptidase domain (yellow) and an HTH motif (violet purple). (**C**) Structural superposition of VapB52^NTD^ and VapB52^CTD^ with their closest structural homologs. (**D**) Structural superposition of VapB52^MD^ with CapP protein from *Thauera* sp. K11 consisting of peptidase domain (wheat), HTH domain (cyan), and GAF domain (blue). (**E**) SAXS scattering profile of the VapBC52 complex. (**F**) Experimental SAXS intensity data (pink circles) fitted with the *ab initio* GASBOR model (black line) (χ² = 2.1). (**G**) Fit of the VapB^Δ23^C52 crystal structure into the SAXS-derived envelope. (**H**) ITC isotherm of VapB52^Δ23^ and VapC52^D6A^ interaction. *K*_D_ and binding stoichiometry are provided in the inset. (**I**) Pull-down assay to assess the role of VapB52 individual domains in binding with VapC52. Abbreviations: P, Pellet; FT, Flow through; W, Wash; E1, Elution 1 (20 mM imidazole); Elution 2 (250 mM imidazole); M, Marker. Labeled marker bands are in kDa. Expected molecular weights of VapB52^ΔNTD^ = 35.6 kDa, VapB52^Δ23,ΔCTD^ = 36.8 kDa and VapB52^MD^ = 29.1 kDa. The panels (H) and (I) are representative of two independent experiments.

To delve into the molecular mechanism of VapC52 neutralization by VapB52, we solved the crystal structure of VapBC52 complex. For co-expression and co-purification of VapC52 and VapB52, 23-residues at the amino-terminus of VapB52 were truncated to remove the predicted disordered region (VapB52^V24-V415^, named VapB52^Δ23^). The crystal structure of VapB^Δ23^C52 complex was determined at a resolution of 3.2 Å by molecular replacement (Fig. 2A and Supplementary Fig. S3A and B). VapB^Δ23^C52 crystallized in the orthorhombic space group C222, with three and six molecules of VapB52^Δ23^ and VapC52 in the asymmetric unit, respectively (Supplementary Fig. S3B). In most VapBC assemblies, VapC dimers adopt a closed conformation with closely positioned active sites, while VapB antitoxins are typically small proteins with structurally distinct N- and C-terminal regions [12, 19, 21, 22, 60, 61]. In contrast to the reported *Mtb* VapB antitoxins (∼8–13 kDa), VapB52^Δ23^ is an unusually large protein (43.25 kDa) consisting of twenty α-helices and four β-strands (Fig. 2B). Structurally, VapB52^Δ23^ is composed of three domains: (i) the N-terminal domain (VapB52^Δ23,NTD^, V24-T93), which comprises of five α helices and binds the outer surface of the VapC52 dimer, away from the active site; (ii) the C-terminal domain (VapB52^CTD^, G357-V415), which comprises of four α helices and occludes the active site of the VapC52; and (iii) the middle domain (VapB52^MD^, T94-A356), which is the largest domain that connects the two terminal domains (Fig. 2B). VapB52^MD^ consists of two sub-domains: (i) peptidase domain: formed by two parallel, two antiparallel β sheets and five α helices with Zn^2+^ bound to conserved HEXXH motif and (ii) HTH motif with four α helices (Fig. 2B and Supplementary Fig. S3C). Comparative structural analysis revealed that VapB52 does not share structural homology with any reported VapB antitoxins. However, the VapB52^Δ23,NTD^ shares structural similarity with other DNA-binding proteins, including C.Csp231I controller of restriction modification system (PDB ID: 4JCY, RMSD = 0.96 Å) [66] and BswR transcription regulator (PDB ID: 4O8B, RMSD = 1.2 Å) [67] (Fig. 2C and Supplementary Table S2). The VapB52^CTD^ shares structural homology with *Staphylococcal aureus* peroxidase inhibitor (PDB ID: 7Z53, RMSD = 2.75 Å) [68] and AvrPtoB effecter from *Pseudomonas syringae* (PDB ID: 3HGK, RMSD = 2.04 Å) [69] (Fig. 2C and Supplementary Table S2). VapB52^MD^ shares structural homology to the CapP protein, which functions as a sensor of fragmented DNA and activates CBASS-mediated phage defense (PDB ID: 7T5T, RMSD = 2.46 Å) [70] (Fig. 2D and Supplementary Table S2), IRRE peptidase from *Deinococcus deserti* (PDB ID: 3DTI, RMSD = 1.83 Å) [71], and *Mtb* PrpR, a transcription regulator (PDB ID: 6D2S, RMSD = 2.3 Å) (Supplementary Table S2) [72]. Interestingly, homologs of the VapB52^MD^ mentioned above have conserved peptidase domain (having Zn^2+^ bound to the HEXXH motif) and DNA-binding HTH motif (Fig. 2D, and Supplementary Fig. S3C and D) [71].

The crystal structure revealed that VapB52^Δ23^ forms a 1:2 stoichiometric heterotrimeric complex with VapC52. This mode of interaction diverges from the classical VapBC complexes, where dimers of VapC and VapB usually interact to form a heterotetrameric (2:2) or hetero-octameric (4:4) assemblies [12, 19, 21, 22, 60, 73, 74]. To further validate the binding stoichiometry observed in the crystal structure, we performed SEC-SAXS experiments for the VapB^Δ23^C52 TA complex (Fig. 2E and Supplementary Fig. S2A–D). The molecular weight of VapB^Δ23^C52 was estimated to be approximately 82.56 kDa (porod volume: 107 330 Å^3^), which was consistent with the theoretical molecular weight of the heterotrimeric VapB^Δ23^C52 complex (82.35 kDa), confirming the oligomeric state observed in the crystal structure. Further, an *ab initio* dummy atom model, based on the SEC-SAXS data, superposed well with the crystal structure of VapB^Δ23^C52 with an NSD value of 2.3 (Fig. 2F and G, and Supplementary Fig. S2F). To further determine the binding affinity of VapC52 and VapB52^Δ23^ isothermal titration calorimetry (ITC) experiments were performed. ITC data revealed binding of VapB52^Δ23^ with VapC52 at a stoichiometric ratio of 1:2 (*N* = 0.44 ± 0.006), further confirming the heterotrimeric state of the complex (Fig. 2H). The dissociation constant of 267 ± 52 nM indicates that the VapB^Δ23^C52 complex has a moderate affinity compared to other VapBC complexes. For instance, VapB11 and VapC11 interact with high affinity with a dissociation constant of ∼ 0.6 nM [12]. PDBePISA analysis (comparing Δ*G*^diss^) of VapBC11, VapBC26, VapBC30, and VapB^Δ23^C52 complexes also suggests that VapB^Δ23^C52 forms a comparatively moderate affinity complex [12, 22, 61] (Supplementary Table S3B).

### VapB52 neutralizes VapC52 *via* a distinct mode of binding

The VapC52 monomers, from the crystal structure of VapC52 and VapBC52 complex, superimposed well with an RMSD of ∼0.72 Å. However, binding of VapB52^Δ23^ with VapC52 brings about a conformational rearrangement mediated by domain motion (maximum cα–cα distance of ∼6.41 Å observed in β3–α7 connecting loop) (Supplementary Fig. S3E). Crystal structure revealed that VapB52^Δ23^ binds the VapC52 dimer at two distinct interfaces (interface I and interface II) that bury a large surface area of 4800 Å^3^ (Fig. 2A and Supplementary Table S3B). The interface I is formed between the VapB52^Δ23,NTD^ and the outer surface of the chain A of VapC52. This interface is stabilized primarily by a multitude of non-bonded interactions, four salt bridges and one hydrogen bond (Supplementary Fig. S3F). Interface II is stabilized by seven hydrogen bonds, two salt bridges, and multiple non-bonded interactions (Supplementary Fig. S3F). Binding of VapB52^CTD^ at the interface II, completely occludes one of the active sites of the toxin dimer (chain A) and partially blocks the accessibility of substrate to the other active site (chain B) (Supplementary Fig. S3G). The complete blocking of one active site while partial masking of the other, and the nature of involvement of these two interfaces in neutralizing VapC52, is unique among VapBC homologs. However, in other VapBC TA complexes, two small VapB antitoxins block the active site of dimeric cognate VapC toxin [12, 19, 21, 22, 60, 61].

To further assess the contribution of different domains of VapB52 in binding with VapC52, we performed pull-down assays using a His-tagged VapC52^D6A^. As shown in Fig. 2I, only VapB52^Δ23,ΔCTD^ was co-purified with VapC52, suggesting that VapB52’s N-terminal domain is critical for VapC52 binding. To assess the role of the zinc binding motif (HEXXH) of VapB52^MD^ in interaction with VapC52, HEXXH was mutated to AEXXA to generate VapB52^H216A,H220A^ (Fig. 2I). The co-purification of VapB52^H216A,H220A^ and His-tagged VapC52^D6A^ indicates that the zinc-binding motif is not required for interaction between VapC52 and VapB52. These data indicate that the VapB52^NTD^, which forms interface I, plays a critical role in toxin binding (Fig. 2A).

### VapB52 self-cleaves in the presence of ssDNA

**Figure 3.**
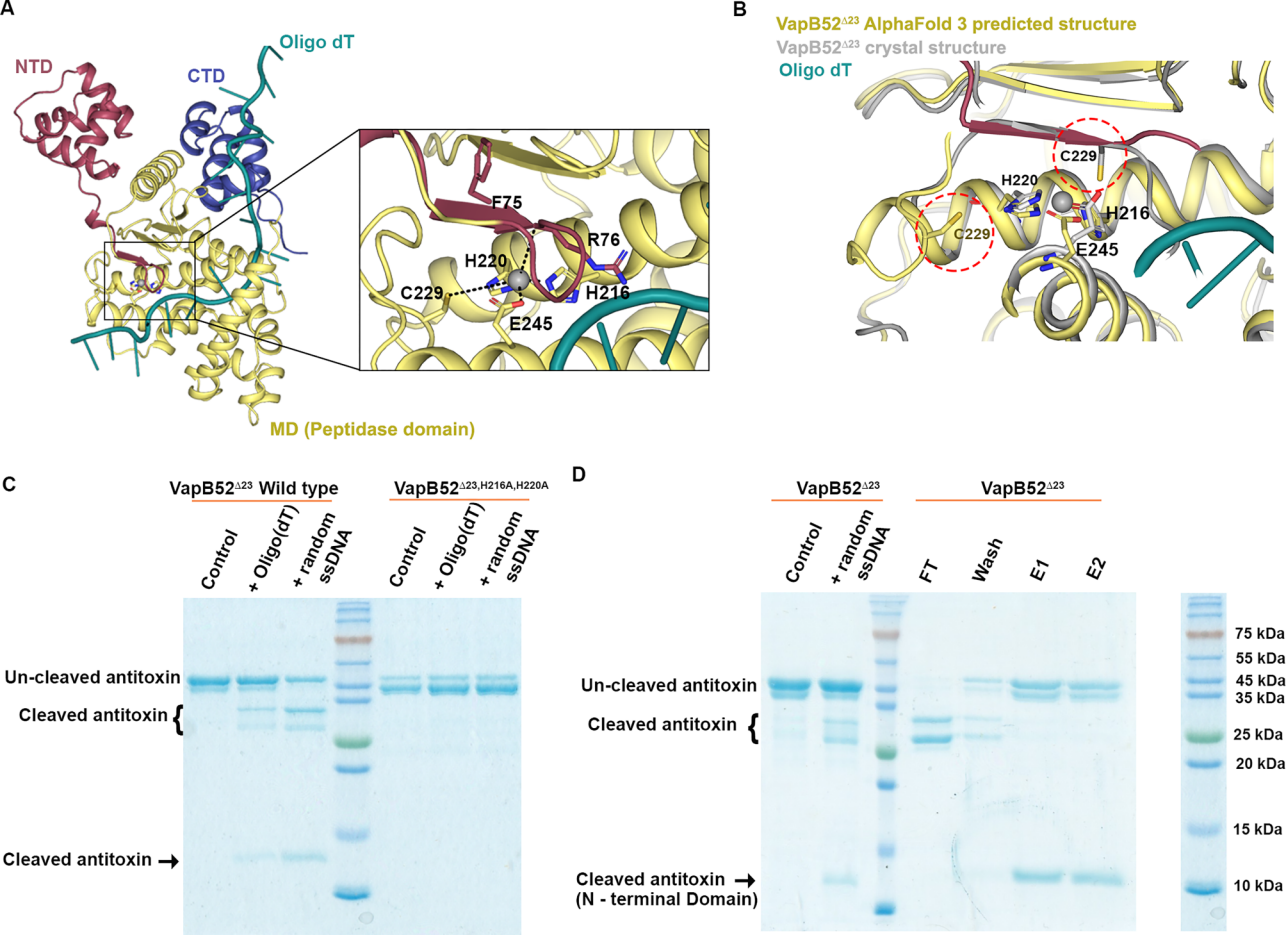
Single-stranded (ss) DNA leads to the self-cleavage of the VapB52^Δ23^. (**A**) Alphafold 3 predicted structure of VapB52^Δ23^ in the presence of oligo(dT) and Zn^2+^ shows the loop connecting NTD and MD positioned in close proximity to the active site residues (H216, H220, and E245). (**B**) Structural superposition revealing conformational rearrangements (dotted red circle) in ‘cysteine switch loop’ in VapB52^Δ23 ^IrrE-peptidase active site. (**C**) SDS–PAGE analysis of ssDNA-induced self-cleavage of VapB52^Δ23^. The wild-type protein (left panel) undergoes cleavage in the presence of oligo(dT) and random single-stranded DNA (as indicated), whereas the active-site mutant shows no detectable cleavage (right panel). (**D**) Ni-NTA purification profile of self-cleaved VapB52^Δ23^ products. A >10 kDa band corresponding to the N-terminal region of VapB52^Δ23^ appeared in the elution fractions E1 (250 mM imidazole) and E2 (500 mM imidazole). The other higher molecular weight (>25 kDa) products appeared in the flow through (FT) and wash fractions. The marker lane indicates the molecular weight standards, with selected band sizes labeled. Data shown in panel (C) and (D) are representative of the two independent biological experiments.

The VapB52^MD^ shares structural similarity with the CapP protein, which harbors the IrrE-family peptidase domain. In the CapP/CapH system, ssDNA binding activates peptidase activity via ‘cysteine switch loop mechanism’. This activated CapP cleaves CapH, subsequently activating Cyclic oligonucleotide-Based Anti-phage Signalling System (CBASS) [70]. Considering structural similarities of VapB52 and CapP, we investigated whether binding of ssDNA activates peptidase activity in VapB52. We predicted the structure of VapB52 in the presence of ssDNA and Zn^2+^ ion using AlphaFold 3 (Fig. 3A). Structural comparison of the crystal structure and AlphaFold 3 generated model of VapB52^Δ23^ revealed notable differences. In the crystal structure, when VapB52 is not bound to ssDNA, Zn^2+^ is coordinated by the H216, H220, E245, and C229, where C229 forms a ‘cysteine switch loop’ and occludes the active site (Fig. 3B and Supplementary Fig. S3C). This conformation closely resembles the inactive state reported for CapP, suggesting that VapB52 within the VapBC52 complex might be similarly auto-inhibited [70]. While in AlphaFold 3 predicted ssDNA bound structure the ‘cysteine switch loop’ exits the active site. This conformational rearrangement renders the active site accessible, thereby activating the IrrE-peptidase domain (Fig. 3B). The conserved F75 and R76 residues in the highly conserved ‘FR motif’, a probable peptidase cleavage site present at the VapB52^NTD^–VapB52^MD^, junction is positioned at the proximity of the peptidase active site (Fig. 3A and Supplementary Fig. S3D). To experimentally validate these predictions, we incubated VapB52^Δ23^ and VapB52^Δ23,H216A,H220A^ active site mutant variant with the ssDNA (24 mer oligo(dT)) and a random primer (24 mer ssDNA) for 30 min, followed by SDS–PAGE analysis. The data revealed that the wild-type VapB52^Δ23^ incubated with oligo(dT) or random primer resulted in self-cleavage (Fig. 3C). However, the active site mutant VapB52^Δ23,H216A,H220A^ did not show any cleavage in the presence of ssDNA (Fig. 3C). To identify the cleaved fragment, we performed Ni-NTA based purification of the cleaved products. The elution fractions showed >10 kDa cleaved fragment and uncleaved VapB52^Δ23^. While the other higher molecular weight (>25 kDa) cleaved bands appeared in the wash fractions (Fig. 3C). Since, the recombinant protein has His-tag at the N-terminus; this data demonstrates that the cleaved fragment corresponds to the VapB52 N-terminal domain.

These findings establish that VapB52 is activated by ssDNA and undergoes self-cleavage *via* a mechanism analogous to the CapP/CapH system. Interestingly, unlike CapP/CapH system, VapB52 undergoes autocatalytic cleavage rather than processing a partner protein. We have already established that the N-terminal domain of VapB52 is crucial for the formation of a stable complex with VapC52 (Fig. 2I). Therefore, autocatalytic cleavage at the NTD/MD linker region of VapB52 likely disrupts VapC52 binding, allowing increased concentration of free toxin in the cell. Collectively, as proposed earlier, these results support a model in which accumulation of ssDNA, arising from DNA damage or phage infection, triggers activation of VapB52, leading to its self-cleavage and increased intracellular concentration of active VapC52 [70]. This provides a mechanistic framework linking environmental stress signals to activation of the VapBC52 TA system.

### Co-expression of non-cognate *Mtb* VapB antitoxins restores the growth defect associated with VapC52 overexpression in *Msm*

**Figure 4.**
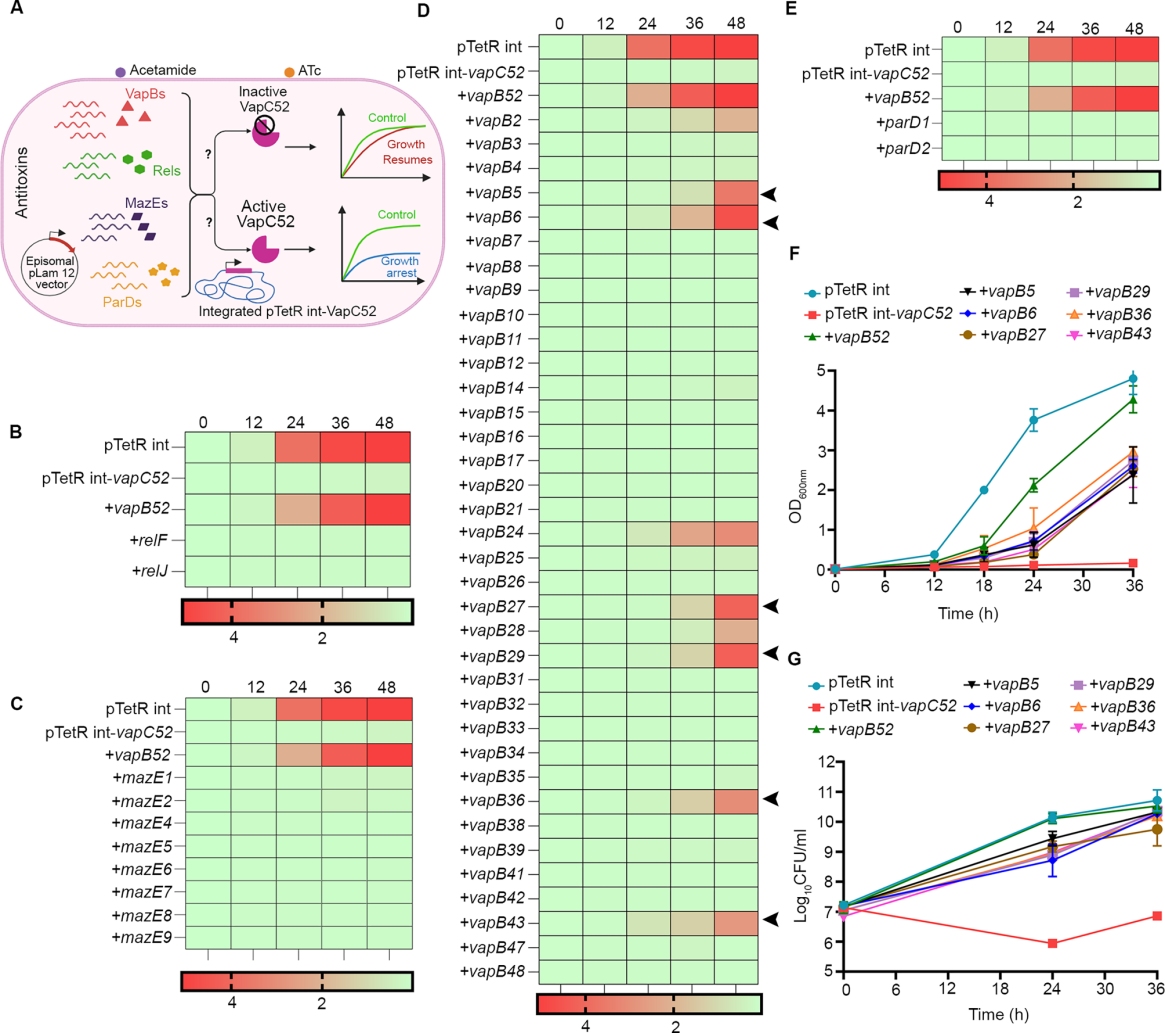
Co-expression of non-cognate VapB antitoxins restores the growth defect associated with VapC52 overexpression in *Msm*. (**A**) Schematic representation of the experimental design for crosstalk experiments between VapC52 and non-cognate antitoxins. (**B–E**) Crosstalk studies performed in *Msm* strain harboring an integrative anhydrotetracycline-inducible copy of vapC52 and Ac-inducible episomal copy of antitoxins relF and relJ (**B**), mazE antitoxins (**C**), vapB antitoxins (**D**), and parD1 and parD2 (**E**). The expression of *vapC52* and recombinant antitoxins in early log phase (OD_600nm_ ∼ 0.2–0.3) in *Msm* cultures was induced by the addition of Atc and Ac. The growth was monitored by measuring OD_600nm_ till the stationary phase. The growth patterns are shown as a heat map. (**F**) Growth patterns of *Msm* harboring integrated copy of *vapC52*, either alone or in the presence of *vapB52, vapB5, vapB7, vapB27, vapB29, vapB36*, and *vapB43*, were monitored by measuring OD_600nm_ at regular intervals. (**G**) For CFU enumeration, ∼10-fold serial dilutions from the cultures (mentioned above) were prepared at time zero, 24 and 36 h post-induction and plated on MB7H11 at 37 °C for 2–3 days. The data shown in this panel are the mean ± SD of log_10_ CFU obtained from two independent experiments performed in duplicates. Panel (**A**) created in BioRender. Singh, H. (2026) https://BioRender.com/ezkhuoe

Earlier studies have established cross-interaction between non-cognate TA systems in various microorganisms such as *E. coli, Mtb*, and *Haemophilus influenza* [23, 24, 29, 75–78]. Presence of an expanded TA repertoire in *Mtb* prompted us to examine cross-interactions between VapC52 and other non-cognate VapB antitoxins [7, 8, 23, 24, 29, 79]. To assess whether VapC52 interacts with non-cognate antitoxins, we performed experiments to determine if co-expression of non-cognate antitoxins restores the growth defect associated with overexpression of VapC52 in *Msm* (Fig. 4A). In addition to VapB52, we found that growth defect associated with overexpression of VapC52 in *Msm* could be restored by co-expressing non-cognate VapB antitoxins such as VapB5, VapB6, VapB27, VapB29, VapB36, and VapB43 (Fig. 4B–E). As shown in Fig. 4F, we observed that overexpression of VapC52 inhibits bacterial growth in a bacteriostatic manner, but co-expression with non-cognate VapB antitoxins such as VapB5, VapB6, VapB27, VapB29, VapB36, or VapB43 restored the growth defect. Consistent with the cell density assays, *Msm* cells co-expressing VapB5, VapB6, VapB27, VapB29, VapB36, or VapB43 demonstrated a marked recovery in CFU counts (Fig. 4G). We also observed that growth defect associated with VapC52 overexpression could not be restored by co-expression of antitoxins from the *relBE, mazEF*, and *parDE* TA systems (Fig. 4B, C, and E). Taken together, these findings imply that in addition to its cognate antitoxin VapB52, growth inhibition observed upon VapC52 overexpression is neutralized by co-expressing a subset of non-cognate VapB antitoxins. These findings expand our understanding of the regulatory networks that exist between VapBC TA systems in *Mtb*.

### VapC52 overexpression reprograms *Mtb* transcriptome and cleaves select *Mtb* tRNAs

**Figure 5.**
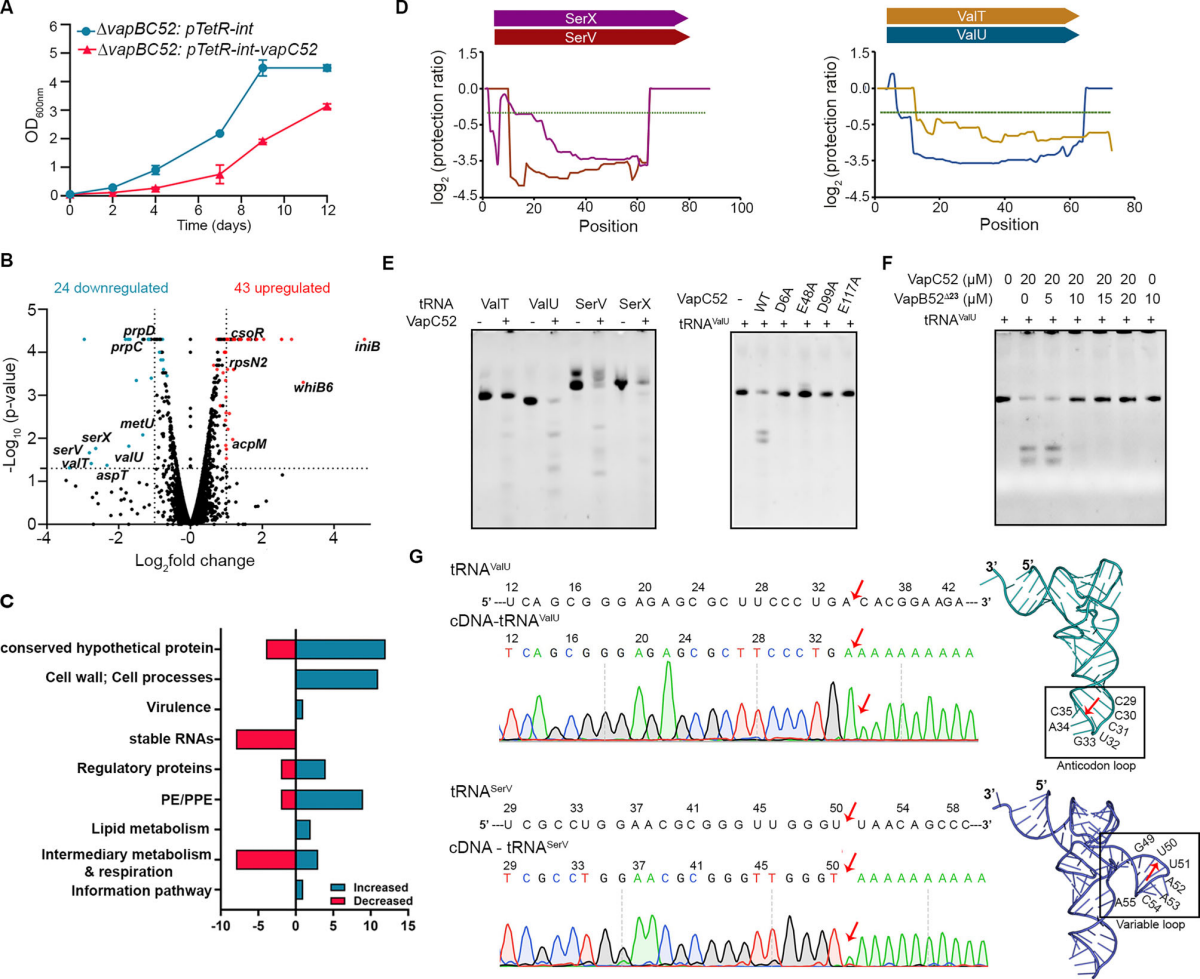
VapC52 cleaves the variable or anticodon loop of the target tRNA. (**A**) The effect of inducible overexpression of VapC52 in early log phase cultures of *ΔvapBC52* was determined by measuring OD_600nm_ at regular intervals till stationary phase was attained. The data shown in this panel are representative of three independent experiments. (**B**) Volcano plot represents differentially expressed genes (DEGs) in *ΔvapBC52* overexpressing VapC52 in comparison to the vector control at 24 h post-Atc induction. The *y*- and *x*-axes depict *P*-value and fold change, respectively. Among these, the relative levels for 43 transcripts (red dots) and 24 transcripts (blue dots), respectively, were differentially expressed. (**C**) Functional categorization of DEGs was based on the Mycobrowser annotation. The *x*-axis represents the number of DEGs per functional category (*y*-axis). The data shown in panels (A) to (C) are obtained from two biological replicates. (**D**) The log_2_ (protection ratio) of tRNA^ValU(GAC)^, tRNA^ValT(CAC)^, tRNA^SerV(GGA)^, and tRNA^SerX(CGA)^ in *ΔvapBC52* overexpressing VapC52 relative to the wild-type strain. (**E**) Urea PAGE confirming VapC52 cleaves down-regulated *Mtb* tRNAs (left panel) while mutant proteins harboring mutations in the PIN domain were inactive in *in vitro* ribonuclease assays (right). (**F**) *In vitro* ribonuclease assays of VapC52 in the presence of different concentrations of VapB52^Δ23^. As expected, complete inhibition of VapC52 ribonuclease activity was observed at a stoichometric ratio of 1:2 (VapB52^Δ23^:VapC52). (**G**) VapC52 cleavage sites in tRNA^ValU(GAC) ^and tRNA^SerV(GGA)^ (left panel), highlighted in AlphaFold 3 predicted tRNA structures (right panel), were verified by DNA sequencing as described in the materials and methods section. The panels (E–G) are representative of two independent experiments.

To investigate the contribution of *vapBC52* TA system in *Mtb* pathophysiology, we constructed Δ*vapC52* and Δ*vapBC52 Mtb* strains using temperature-sensitive mycobacteriophages (Supplementary Fig. S4A and B). The construction of Δ*vapC52* and Δ*vapBC52 Mtb* strains was verified by Southern blotting using locus-specific probes (Supplementary Fig. S4C and D). We noticed that deletion of either *vapC52* or *vapBC52* did not affect *Mtb*’s ability to form biofilms, colony morphology or growth in liquid medium (Supplementary Fig. S4E and F). Collectively, these data suggest that the VapBC52 TA system is dispensable for the *in vitro* growth of *Mtb* in both solid and liquid media. Previously, it has been shown that toxin overexpression reprograms bacterial transcriptional and metabolomic profiles, thereby promoting stress adaptation [8, 12, 16, 23, 26, 28, 29, 80–83]. As shown in Fig. 5A, anhydrotetracycline inducible overexpression of VapC52 inhibited the growth of *ΔvapBC52 in vitro*. Next, we compared the transcriptional profiles of *ΔvapBC52* harboring either vector or pTetR-int-*vapC52* to determine the cellular target of VapC52. Using a cut-off of ± 2.0-fold and *P*-value of < 0.05, we found that the relative levels of 43 and 24 transcripts were increased or decreased, respectively, in VapC52 overexpression strain compared to the vector control strain (Fig. 5B). More detailed analysis of the RNA-seq data revealed that the transcripts with higher relative levels in VapC52 overexpression strain encoded for either conserved hypothetical proteins or proteins involved in cell wall biosynthesis and cellular processes (Fig. 5C). The majority of transcript levels with reduced relative levels encoded for stable RNAs (sRNAs) and genes involved in intermediary metabolism and respiration (Fig. 5C and Supplementary File S2).

Next, we calculated log₂ protection ratios at single-nucleotide resolution to quantify the relative susceptibility of tRNAs to VapC52-mediated cleavage as previously reported [23]. Using a threshold of log₂ (protection ratio) ≤ −2.5, we observed a substantial decrease in protection for a number of tRNAs, including tRNA^ValT(CAC)^, tRNA^GlyU(CCC)^, tRNA^AsnT(GTT)^, tRNA^HisT(GTG)^, tRNA^AlaU(GGC)^, tRNA^ValU(GAC)^, tRNA^GluU(CTC)^, tRNA^SerV(GGA)^, tRNA^SerX(CGA)^, and tRNA^SerU(TGA)^ in the VapC52-overexpressing strain compared to the parental control (Fig. 5D, and Supplementary Table S4 and Supplementary File S3). These findings suggest that overexpression of VapC52 inhibits bacterial growth by cleaving several tRNAs. Therefore, we speculated that these tRNAs might be the *in vitro* cellular target for VapC52. Next, we performed *in vitro* ribonuclease assays and observed that VapC52 cleaves *Mtb* tRNAs (tRNA^ValU(GAC)^, tRNA^ValT(CAC)^, tRNA^SerV(GGA)^, and tRNA^SerX(CGA)^) (Fig. 5E, left panel). As anticipated, VapC52 harboring mutations in PIN domain D6, E48, D99, and E117 were unable to cleave tRNA^ValU(GAC)^, highlighting the importance of these residues in ribonuclease activity (Fig. 5E, right panel). Additionally, we performed VapC52 tRNase assays in the presence of varying concentrations of VapB52^Δ23^. In agreement with our earlier observations, binding of VapB52^Δ23^ completely inhibited VapC52 activity when present at a stoichometric ratio of at least 1:2, respectively (Fig. 5F). As shown in Fig. 5E, VapC52-mediated cleavage of tRNA^ValU(GAC)^ and tRNA^SerV(GGA)^ resulted in two and three cleavage products, respectively. Furthermore, to map the cleavage site for tRNA^VaUl(GAC)^ and tRNA^SerV(GGA)^ by VapC52, we performed reverse transcription followed by DNA sequencing of the cleaved fragments as described in Materials and methods. DNA Sanger sequencing data confirmed that VapC52 cleaves between A34↓C35 site corresponding to the anticodon loop region of tRNA^ValU(GAC)^ and between the U51↓U52 variable loop tRNA synthetase binding region of tRNA^SerV(GGA)^ (Fig. 5G). To the best of our knowledge, this is the first report of VapC toxin-mediated cleavage of a functionally relevant variable loop region in tRNA. Taken together, these observations demonstrate that VapC52 overexpression inhibits bacterial growth by cleaving functionally important loop regions in the tRNAs.

### VapBC52 is required for *Mtb* to establish infection in guinea pigs

**Figure 6.**
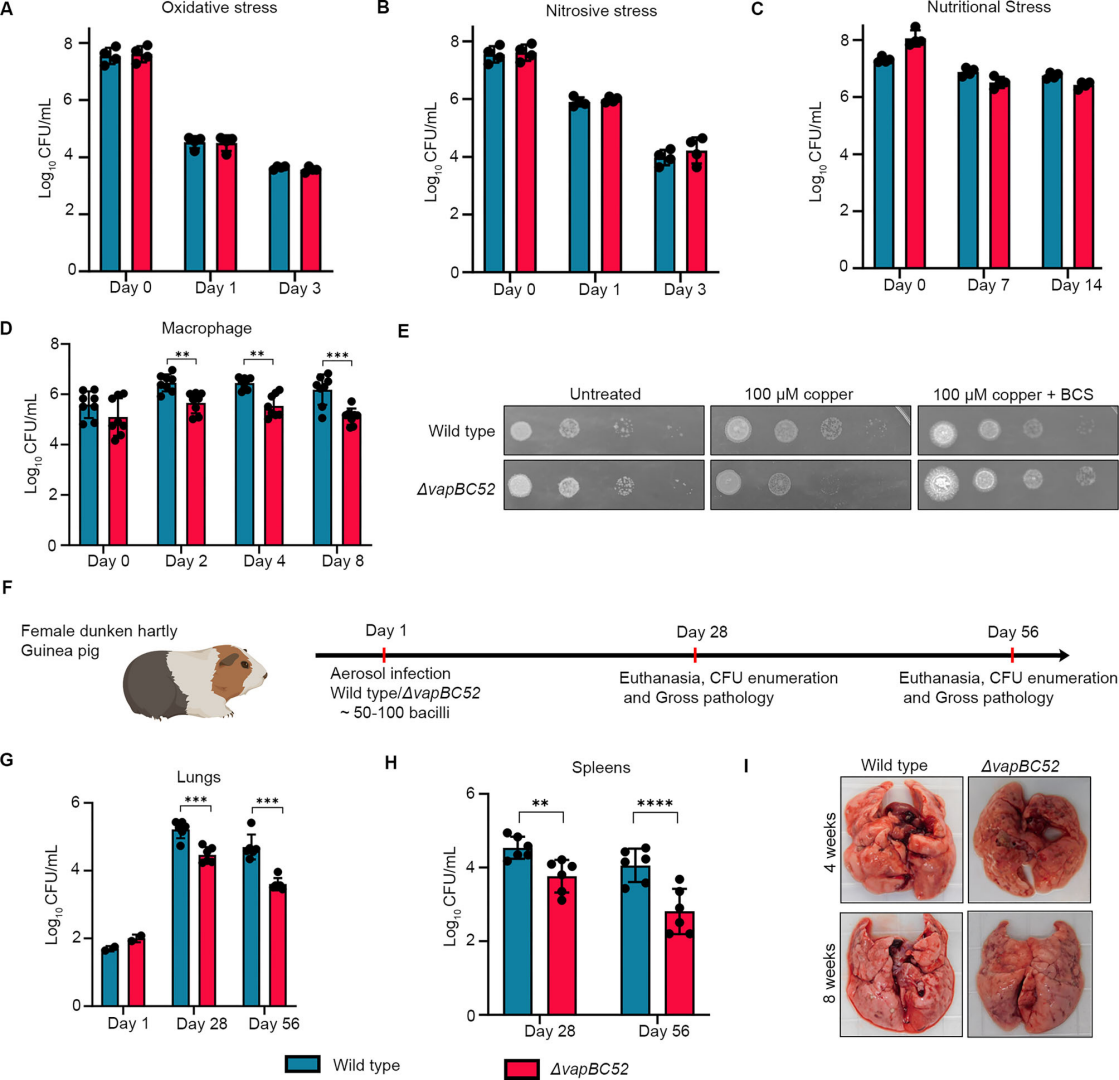
VapBC52 is required for copper tolerance and establishment of disease in guinea pigs. (**A–C**) Early log phase cultures (OD_600nm_ ∼ 0.2–0.3) were exposed to oxidative stress (5 mM H_2_O_2_, A), nitrosative stress (5 mM NaNO_2_, B), or nutritional stress (1× TBST, C) as described in Materials and methods. Bacterial counts were determined, and data represent mean ± SD of log_10_ CFU obtained from two independent experiments performed in duplicate or triplicate. The day zero readings shown in panels (A) and (B) are derived from the same experiment. (**D**) THP1 macrophages were infected with either wild-type or *ΔvapBC52 Mtb* at MOI 1:10, and the survival of intracellular bacteria was determined at designated time points as described in Materials and methods. The data presented in this panel is the mean ± SD of log_10_ CFU obtained from three independent experiments performed in triplicate or duplicate. (**E**) Growth of either parental or *ΔvapBC52 Mtb* was measured in MB7H11 medium in the absence or presence of copper and BCS by spotting assays as described in the Materials and methods. The data shown in these panels is representative of three independent experiments. (**F**) Guinea pigs were infected with either wild-type or *ΔvapBC52* strains of *Mtb* to establish ∼50–100 bacilli in the lungs. The extent of disease progression was determined at 4- and 8– weeks post-infection. (**G** and **H**) The data shown in these panels is mean ± SD of log_10_ CFU obtained in lungs (G) and spleens (H) from guinea pigs (*n* = 5–6) at 4- and 8 weeks post-infection. (**I**) Representative gross pathology images of lungs from infected guinea pigs at 4- and 8 weeks (*n* = 5–6 per group) are shown in this panel. The data shown in panels (D), (G), (H), and (I) are representative of two independent experiments. Statistical significance determined by unpaired *t*-test (**P-value < 0.01, ***P value < 0.001, ****P-value < 0.0001).

Numerous studies have demonstrated that *Mtb* adapts and persists within the host despite exposure to unfavourable conditions such as reactive oxygen species, nutrient starvation, low oxygen or acidic pH [18, 24, 25, 28, 84–88]. It has also been shown that a subset of toxins and antitoxins is differentially expressed in *Mtb* upon exposure to distinct stress stimuli and in infected macrophages [8, 25, 80, 83, 89]. TA systems from various organisms have been implicated in disease pathogenesis [12, 18, 25–28, 30, 75, 90–93]. Here, we also compared the survival of wild-type and Δ*vapBC52* upon exposure to stress conditions that the bacteria might encounter in host tissues. We observed that deletion of *vapBC52* did not impair the survival of *Mtb* upon exposure to oxidative, nitrosative and nutritional stress (Fig. 6A–C). To delineate the contribution of VapBC52 in intracellular survival of *Mtb*, we compared the survival of wild-type, Δ*vapC52* and Δ*vapBC52* in THP-1 macrophages. We found that deletion of *vapBC52* reduced *Mtb* growth by ∼ 5.0-fold, ∼ 5.0-fold, and ∼9.0-fold, respectively, at days 2, 4, and 6 post-infection (**P < 0.01, ***P < 0.001) (Fig. 6D). However, the growth of Δ*vapC52* and parental strain was similar in THP-1 macrophages at days 2, 4, and 6 post-infection (Supplementary Fig. S5A). These findings suggest that deletion of *vapBC52* locus impaired the intracellular survival of *Mtb* in THP1 macrophages.

It has been shown that overexpression of VapC4 and VapC36 in *Mtb* induces the expression of genes involved in copper tolerance [16, 75]. We also observed that the relative levels of *csoR* (copper-sensing operon repressor) were higher in the *Mtb* strain overexpressing VapC52 in comparison to the vector control strain (Supplementary File S2). It is well established that macrophages increase the phagosomal copper concentration upon *Mtb* infection [94–97]. We have recently reported that deletion of a subset of the *vapBC* TA system enhances *Mtb’s* susceptibility to copper [75]. Therefore, we also compared the sensitivity of wild-type, Δ*vapC52*, and Δ*vapBC52* strains in copper-supplemented 7H11 medium. As shown in Fig. 6E, Δ*vapBC52* showed increased susceptibility upon exposure to copper relative to the wild-type strain. To verify the specificity of this growth defect, we repeated spotting assays in the presence of bathocuproinedisulfonic acid disodium salt (BCS), a highly selective bidentate chelator of monovalent copper (I) ions [98, 99]. The restoration of the growth defect associated with Δ*vapBC52* in the presence of BCS suggests that this is a copper-specific effect (Fig. 6E). Additionally, we observed that the growth patterns of the wild-type and Δ*vapC52* were similar in copper-supplemented 7H11 medium (Supplementary Fig. S5B).

Previously, it was reported that the copper-sensitive *mctB* mutant strain exhibited a more pronounced phenotype in guinea pigs relative to mice [99]. This difference has been attributed to the increased bactericidal activity of copper under low-oxygen conditions, where it predominantly exists in the more toxic reduced cuprous (Cu^+^) state. To delineate the role of VapBC52 in *Mtb* pathogenesis, we compared the growth of wild-type and Δ*vapBC5*2 in aerosol infected guinea pigs (Fig. 6F). The bacillary loads decreased by ∼6.0-fold and ∼ 12.0-fold at 4 and 8 weeks post-infection, respectively, in lungs of Δ*vapBC52* infected guinea pigs strain compared to wild-type infected guinea pigs (***P < 0.001) (Fig. 6G). As shown in Fig. 6H, the splenic bacillary loads in the guinea pigs infected with the Δ*vapBC52* strain were significantly reduced by ∼12.0-fold and ∼36.0-fold at 4 and 8 weeks post-infection compared to guinea pigs infected with the wild-type strain (**P < 0.01, ****P < 0.0001). In concordance with macrophage data, we observed that the lungs and splenic bacillary loads in wild-type and Δ*vapC52* infected guinea pigs were comparable at both 4 and 8 weeks post-infection (Supplementary Fig. 5C–F). In agreement with CFU data, we observed less tissue damage in the lung tissues of guinea pigs infected with Δ*vapBC52* compared to animals infected with the wild-type strain (Fig. 6I). Overall, these findings imply that the VapBC52 TA system contributes to copper tolerance and disease pathogenesis of *Mtb*.

### VapC52 cleaves phage tRNAs and contributes to defense against mycobacteriophages

**Figure 7.**
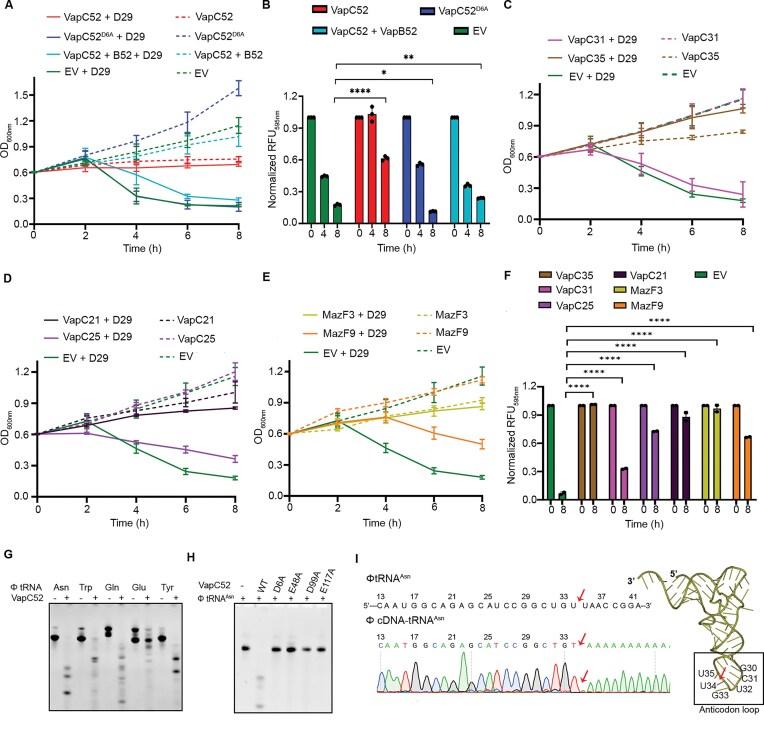
VapC52 provides antiphage defense in *Msm* against mycobacteriophage D29. (**A**) Growth curves of *Msm* overexpressing VapC52, VapC52^D6A^ or co-expressing VapC52 along with VapB52, with and without D29 infection. The data shows that *Msm* overexpressing VapC52 is able to restore the growth upon D29 infection, indicating that overexpressing VapC52 protects *Msm* from D29-mediated killing. (**B**) Alamar blue assay performed to check the cell viability of D29-infected *Msm* cells expressing VapC52, VapC52^D6A^, and co-expressing VapC52 along with VapB52. Statistical significance determined by two-way ANOVA (**P-value < 0.01, ***P-value < 0.001, ****P-value < 0.0001). (**C** and **D**) Growth curve analysis showing the effect of VapC31(C), VapC35(C), VapC21(D), and VapC25 (D) toxin overexpression in *Msm* in the absence (dotted lines) or presence (solid lines) of D29 phage infection. (**E**) Growth curve analysis showing the effect of MazF3 and MazF9 overexpression in *Msm* in the presence (solid lines) or absence (dotted lines) of the D29 infection. (**F**) Alamar blue, cell viability assay of *Msm* overexpressing the selected toxins such as VapC35, VapC31, VapC25, VapC21, MazF3, and MazF9 followed by phage infection, shows that VapC35, VapC25, VapC21, and MazF3 and MazF9 provides phage defense through abortive infection. Data in panels (A–F) represent the mean of triplicate measurements from two independent experiments. The data shown in panels (A–F) are representative of two independent experiments. Empty Vector (EV) data used in panels (C–E) is derived from the same experiment. Statistical significance determined by two-way ANOVA (**P-value < 0.01, ***P-value < 0.001, ****P-value < 0.0001). (**G**) Urea PAGE analysis shows VapC52 cleaves all five D29 encoded tRNAs [tRNA^Asn(GTT)^, tRNA^Trp(CCA)^, tRNA^Gln(CTG)^, tRNA^Glu(CTC)^ and tRNA^Tyr(GTA)^]. (**H**) tRNA cleavage assay showing that PIN domain mutants of VapC52 are unable to cleave phage encoded tRNA^Asn(GTT)^. (**I**) DNA Sanger sequencing reveals VapC52 cleaves site between U34↓U35 position located in the anticodon loop of tRNA^Asn(GTT)^. The data shown in panels (**G**) and (**H**) are representative of two independent experiments.

TA systems are known to provide resistance to bacteria against phage infection [31, 32, 100]. Zhang *et al*., showed that deletion of all eight TA systems increases susceptibility of *Msm* to phage infection [101]. To understand whether VapC52 contributes to phage defense in mycobacteria, we used *Msm* as a model. *Msm* harboring vector control and toxin overexpression plasmid were infected with mycobacteriophage D29 (Fig. 7A). We noticed that *Msm* expressing VapC52 was able to survive D29 phage-mediated killing (Fig. 7A). In comparison, *Msm* overexpressing VapC52^D6A^ and co-expressing VapC52 along with VapB52 showed a drastic decrease in bacterial growth upon D29 infection (Fig. 7A). Taken together, these observations suggest that VapC52 confers protection against phage infection in *Msm*.

It is known that bacteria deploy abortive infection as a defense strategy against phages [31, 102]. In this process, the phage-infected cells die prematurely, thus preventing the completion of the phage lifecycle, thereby protecting the neighbouring cells and the bacterial population from phage infection [34, 102]. To investigate if VapC52 provides anti-phage defense through abortive infection, we determined bacterial viability in infected *Msm* cultures using Alamar blue assay. We observed that upon D29 mycobacteriophage infection, *Msm* overexpressing VapC52 was moderately viable, whereas VapC52^D6A^ and VapB52–VapC52 co-expressing cells showed a drastic reduction in the number of viable bacteria after 8 h of D29 mycobacteriophage infection (Fig. 7B). These findings imply that VapC52 contributes to antiphage defense through abortive infection. Further, we performed experiments to determine whether other *Mtb* toxins also contribute to antiphage defense in *Msm*. To investigate this further, we selected representative toxins from the VapC (VapC35, VapC31, VapC25, and VapC21) and MazF (MazF3 and MazF9) families to assess their role in antiphage defense (Fig. 7C–E). Based on growth curve analysis, we conclude that in addition to VapC52, VapC35, VapC21, and MazF3 also contribute to antiphage defense in *Msm* (Fig. 7C–E). In Alamar blue assays, we observed that the viable bacterial counts were marginally reduced in *Msm* expressing VapC35, VapC25, VapC21, MazF9, and MazF3 upon D29 infection. Taken together, our findings imply that a subset of these TA systems confer antiphage defense by abortive infection in *Msm* (Fig. 7F).

Given that VapC52 inhibits *Mtb* growth by cleaving tRNA, we speculated that VapC52 contributes to antiphage defense by cleaving D29 mycobacteriophage-encoded tRNAs. To verify this, we examined the ribonuclease activity of VapC52 against the five D29 phage-encoded tRNAs viz. tRNA^Asn(GTT)^, tRNA^Trp(CCA)^, tRNA^Gln(CTG)^, tRNA^Glu(CTC)^, and tRNA^Tyr(GTA)^. We observed that VapC52 was able to cleave phage-encoded tRNAs (Fig. 7G). As anticipated, phage-encoded tRNA^Asn(GTT)^ could not be cleaved by VapC52 variants harboring mutations in the PIN domain (Fig. 7H). DNA sequencing further confirmed that VapC52 cleaves tRNA^Asn(GTT)^ between the anticodon region’s U34 and U35 position (Fig. 7G). To the best of our knowledge, this is the first study providing insights into the mechanism by which VapC toxins from the VapBC TA system contribute to antiphage defense by cleaving the phage-encoded tRNAs.

## Discussion

This study unravels several unique structural features and functional roles of the VapBC52 TA system from *Mtb*. Notably, VapB52 is an exceptionally large antitoxin (∼46 kDa) compared to VapB proteins (~8–13 kDa) that belong to other *Mtb* VapBC TA systems. Consistent with this, our findings revealed an unusual domain arrangement in VapB52 and a heterotrimeric oligomeric state of VapBC52 complex. Thus, VapB52’s multidomain structure seems to compensate for the homodimeric nature of canonical VapB proteins, enabling effective binding to VapC52 dimer. In contrast to closed symmetric assemblies, as reported for other VapC structures, VapC52 itself adopts an ‘open’ dimeric and flexible conformation [11, 12, 19–22, 61, 64]. These structural distinctions underpin the unique regulatory mechanism of VapBC52 and contribute to the architectural diversity that exists among the VapBC subfamily of TA systems. We also observed that co-expressing VapB52 and several non-cognate VapB antitoxins restored the growth inhibition associated with VapC52 overexpression in *Msm*. This promiscuity in antitoxin binding suggests that VapBC52 may function as part of a broader regulatory network, rather than an isolated TA system. Such plasticity in VapBC TA system interactions might enable *Mtb* to fine-tune growth, survival, and defense strategies in dynamic host environments.

**Figure 8.**
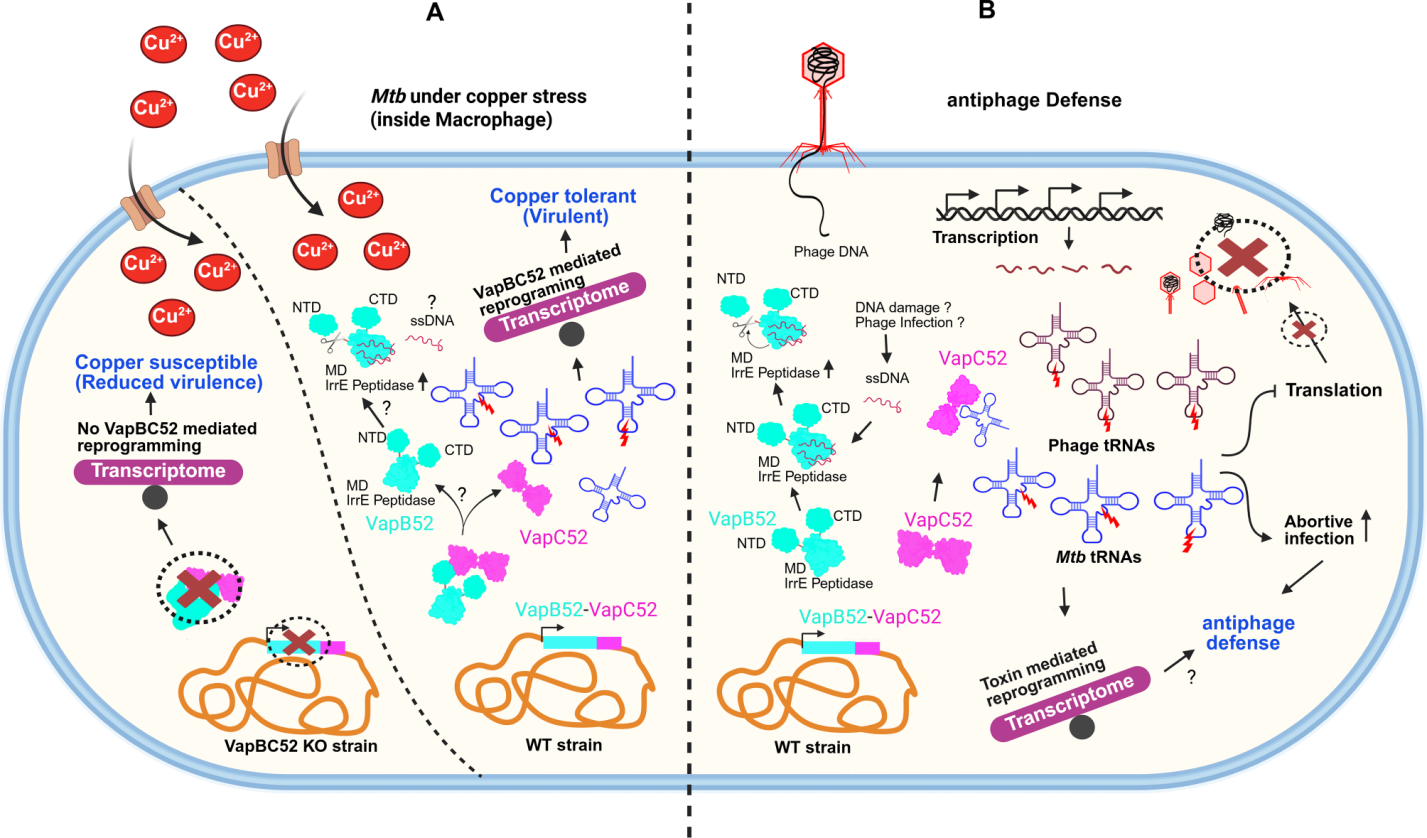
Proposed role of VapBC52 TA system in copper tolerance, *in vivo* survival and phage defense. The levels of intracellular copper increase in the presence of intracellular pathogens like *Mtb. Mtb* has evolved several strategies to survive in these high intracellular copper levels. We hypothesize that in the presence of copper (**A**), or phage infection (**B**), the presence of ssDNA activates the cysteine switch loop in VapB52, resulting in self-cleavage of VapB52^NTD^. This N-terminal domain in VapB52 is crucial for toxin binding and neutralization. The presence of free toxin slows down bacterial growth by cleaving *Mtb* tRNAs [tRNA^ValU(GAC)^, tRNA^ValT(CAC)^, tRNA^SerV(GGA)^, and tRNA^SerX(CGA)^] (**A**) and/or phage encoded tRNAs (tRNA^Asn(GTT^), tRNA^Trp(CCA)^, tRNA^Gln(CTG)^, tRNA^Glu(CTC)^, and tRNA^Tyr(GTA)^] (**B**) at anticodon or variable loop regions (red thunder sign). This VapC52-mediated tRNA cleavage results in transcriptional reprogramming that enables *Mtb* to survive in the presence of copper and host tissues (**A**). Further, we also propose that cleavage of phage-encoded tRNAs by VapC52 inhibits phage propagation by an abortive infection (Abi) mechanism. This is the first study demonstrating the role of VapBC52 TA system in *Mtb* pathogenesis and phage defense in *Msm*. Created in BioRender. chaudhari, V. (2026) https://BioRender.com/lc93kb7.

In the present study, we also demonstrate that *vapBC52* locus is dispensable for *in vitro* growth but is essential for survival under copper stress, within macrophages, and during infection (Fig. 8A). *Mtb* faces high copper concentrations in host phagosomes as part of the innate immune response [94, 95, 99, 103]. VapC4 has been shown to mediate copper resistance by inducing sulfur metabolic pathways via targeting tRNA^Cys (GCA)^ [16, 75]. The requirement of VapBC52 for intracellular survival underscores its relevance to *Mtb* virulence, adding to the growing body of evidence that TA systems contribute directly to pathogenesis beyond stress adaptation. In agreement with previously published reports, we observed that, similar to overexpression of VapC or MazF homologs, the overexpression of VapC52 in *Mtb* also resulted in transcriptional reprogramming [17, 23, 25, 29, 81]. The detailed analysis of RNA-seq data revealed that VapC52 overexpression strain had relatively lower levels of numerous tRNAs than the vector control strain. Concurrent with this observation, we show that VapC52 cleaves these tRNAs at functionally relevant anticodon or variable loop regions. We propose that this VapC52-mediated tRNA cleavage results in transcriptional reprogramming and reduced bacterial growth.

The antiphage defense systems in bacteria and phage defense systems targeting bacteria are continuously evolving [37]. While bacteria code for antiphage defense modules to defend themselves from incoming phages, the latter also evolve to overcome such barriers to successfully infect the host. Bacterial TA systems are emerging as important antiphage defense systems. Several studied cases show that upon phage attack, many bacterial-encoded toxins are activated, which disrupt the viral propagation by depleting the tRNA pool *via* targeted cleavage of the host tRNAs [104, 105]. To overcome this defense mechanism, many bacteriophages encode their own tRNAs [106–108]. In this study, we show that overexpression of VapC52 in *Msm* makes the bacterium resistant to D29 mycobacteriophage infection. Our data suggest that the antiphage defense in *Msm* upon VapC52 overexpression occurs due to the cleavage of phage-encoded tRNAs (Fig. 8B). These findings add VapBC52 to the growing repertoire of antiphage defense systems. In addition to VapC52, we also identified VapC35, and VapC21 belonging to VapBC TA system and MazF3 belonging to MazEF TA systems that conferred protection against phage infection in both growth curve and Alamar blue assays. This reinforces the concept that multiple, mechanistically distinct TA toxins can indeed provide antiphage defense by likely targeting different stages of the phage life cycle. Nevertheless, given the targeted cleavage of phage-encoded tRNAs and the highly conserved mycobacterial tRNAs, we are tempted to suggest that VapC52 could also contribute to antiphage defense in slow-growing and clinically important mycobacteria such as *Mtb*. However, experimental evidence in this regard is currently lacking. Future experiments would be performed to determine the role of VapC52, VapC35, VapC21, and MazF3 in antiphage defense for various clinical mycobacterial strains.

Interestingly,VapB52 also shares structural homology with the CapP protein from *Thauera* sp. K11. Notably, CapP protein has a peptidase domain that degrades the repressor of activators of the antiphage defense system, while its HTH motif senses the fragmented DNA during the phage infection [70]. High structural homology between VapB52 and CapP-like M domain suggests a potential role of VapB52 as a sensor or modulator in response to phage infection. In CapP/CapH system, CapP cleaves CapH and activates CBASS anti phage defense system [70]. Our data and published report suggest that both CapP and VapB52 probably share proposed ‘cysteine switch loop’ mechanism [70]. Also, VapB52 has a unique three domain architecture and harbors highly conserved ‘FR’ sequence motif, and undergoes self-cleavage in the presence of ssDNA. This self-cleavage likely disrupts toxin-binding ability/neutralization activity of VapB52, resulting in excess free active VapC52 toxin and subsequent downstream effects. Interestingly, the roles of both CapP (IrrE peptidase activity) and CapH (substrate) are present in VapB52 itself. Considering the growing interest in alternative therapies, including phage therapy [109] due to the emergence of antibiotic-resistant pathogens, the study of antiphage defense mechanisms and their counter-mechanisms in bacteriophages will guide the engineering of more efficient and effective phages with therapeutic potential.

In conclusion, VapBC52 represents a structurally distinct and functionally versatile TA system in *Mtb*. VapC52 is a tRNase that inhibits *Mtb* growth by specifically cleaving tRNAs in functional loop regions. Structure determination studies revealed that VapC52 adopts a unique open-dimer conformation and is asymmetrically neutralized by a large multi-domain antitoxin, VapB52. We further show that in the presence of ssDNA, VapB52 is auto-cleaved. We hypothesize that this auto-cleavage of VapB52 may be associated with the activation of VapBC52 TA system. We also show that VapBC52 TA system is dispensable for *in vitro* growth but contributes to copper tolerance, disease pathogenesis, and is likely involved in antiphage defense. Collectively, the results presented in this study significantly expand our understanding about the structural and functional regulation of TA systems.

## Supplementary Material

gkag611_Supplemental_Files

## Data Availability

The crystal structure coordinates and structure factor file have been deposited in RCSB PDB under the PDB DOIs: https://doi.org/10.2210/pdb9vzy/pdb and https://doi.org/10.2210/pdb9vzz/pdb. The Bio-SAXS data are deposited at SASDB (https://www.sasbdb.org/) with accession codes: SASDX27 and SASDX37. RNA-Seq data have been deposited in the NCBI Gene Expression Omnibus (GEO, https://www.ncbi.nlm.nih.gov/geo/) under accession number GSE308059.
