# Supplementary material for: Structural and functional characterization of VapBC52 toxin–antitoxin system from Mycobacterium tuberculosis
Source: Nucleic Acids Res. 2026 Jun 17;54(11):gkag611. doi: 10.1093/nar/gkag611 (PMC13273310; doi:10.1093/nar/gkag611)
Supplement: gkag611_Supplemental_Files [file gkag611_supplemental_files.zip › Supplementary data revised.doc]

**Supplementary Data**

**Structural and functional characterization of VapBC52 toxin–antitoxin system from *Mycobacterium tuberculosis***

Manisha Singh^1#^, Charandeep Singh^2,3#^, Akshay V Nair^4^, Imran Ahmad^1,5^, Arun Sharma^1^, Munmun Bhasin^6^, Vikas Jain^4,7^, Ramandeep Singh^1^*, Krishan Gopal Thakur^2,3,8^*.

**^1^** Centre for Tuberculosis Research, Tuberculosis Research Laboratory, BRIC-Translational Health Science and Technology Institute, Faridabad-Gurugram expressway, Faridabad, Haryana, India.

**^2^**Structural Biology Laboratory, CSIR-Institute of Microbial Technology, Sector 39A, Chandigarh - 160036, India.

**^3^** Academy of Scientific and Innovative Research (AcSIR), Ghaziabad - 201002, India.

**^4^**Microbiology and Molecular Biology Laboratory, Department of Biological Sciences, Indian Institute of Science Education and Research (IISER) Bhopal, Madhya Pradesh, 462066, India

**^5^**Manipal Academy of Higher Education, Manipal, Karnataka 576104, India.

**^6^**Molecular Biophysics Unit, Indian Institute of Science, Bangalore-560012, India.

**^7^**Laboratory of Antimicrobial Innovation, School of Interwoven Arts and Sciences, Krea University, Sri City – 517646, Andhra Pradesh, India.

**^8^**Department of Biotechnology, National Institute of Pharmaceutical Education & Research (NIPER), Sector 67, S.A.S. Nagar - 160062, Punjab, India.

- **^#^**These authors contributed equally
- *****Correspondence
- ramandeep@thsti.res.in, krishang@niper.ac.in

**Key words;** *Mycobacterium tuberculosis*, Toxin-antitoxin system, virulence associated protein B and C, tRNase, pathogenesis, phage defence

.

**Supplementary Figure 1.** Crystal structure reveals the unique dimeric interface of VapC52^D6A^. **(A)** Alignment of VapC52 with closely related sequences highlighting highly conserved catalytic residues (D6, E49, D99, and E117) characteristic of PIN and DUF4411 domain containing proteins (asterisks). **(B)** Purification of VapC52 using SEC shows a predominant peak and SDS PAGE confirms the expected molecular weight (~20 kDa). **(C)** Asymmetric unit (ASU) contains four molecules of VapC52, represented in putty style, where tube thickness represents B-factors. Chains A and B show low B factors (less thermal motion) while chains C and D show high B-factors (higher flexibility). (**D)** Electrostatic potential map (two orientations) highlights that active sites (circles) of toxins in dimeric assembly face away from each other. **(E)** Dimeric interface of VapC52^D6A^ with two-fold symmetry (left) and close-up view of the residues forming hydrogen bonds at the binding interface (right)**. (F)** Hydrogen bonds and salt bridges stabilizing the dimeric interface between the VapC52 monomer are shown in blue and cyan colors, respectively.

**Supplementary Figure 2.** Solution structure of VapC52^D6A^ toxin and VapB^Δ23^C52 TA complex**. (A)** SEC-SAXS intensity profile of VapC52 (blue) and VapB^Δ23^C52 (pink) showing a single predominant peak, indicating a homogeneous population with Rg value ~2.39 nm for VapC52 (blue) and Rg value ~3.14 nm for VapBC2 (pink). (**B)** Normalized pair distribution function P(r) plot showing the maximum dimensions of protein molecules in solution, VapC52 with D_max_ of 8.5 nm (blue) and VapBC52 with D_max_ of 10.4 nm (pink). (**C)** Dimensionless Kratky plot adopts bell shaped curve for both VapC52 (blue) and VapB^Δ23^C52 (Pink) a characteristic feature of a compact, globular and well-folded conformation of proteins in solution. **(D)** Guinier analysis of VapC52^D6A^ (blue) and VapB^Δ23^C52 (pink) with linear fit reveals Rg of 2.39 nm and 3.14 nm, respectively. **(E)** Dimeric VapC52 crystal structure fits into *ab initio* GASBOR models with NSD = 2.5 using SUPALM. **(F)** Hetero-trimeric crystal structure of VapB^Δ23^C52 fits into *ab initio* dummy atom model NSD = 2.7 using SUPALM.

**Supplementary Figure 3**. Crystal structure of VapB^Δ23^C52 complex. **(A)** Size-exclusion chromatogram of co-purified VapB^Δ23^C52 complex showing a single peak. SDS–PAGE analysis reveals two bands corresponding to the toxin (~20 kDa) and antitoxin (~43 kDa). **(B)** Asymmetric unit (ASU) contains three antitoxin and six toxin molecules arranged in 1:2 stoichiometry, respectively. Most residues are well resolved, with missing electron densities in flexible or disordered regions. In antitoxin chains A–C, unresolved residues include G82–A83 and E310–G335 (A); L78–S83, V309–G335, and S390–V392 (B); and L78–A81, V309–G336, and S390–V392 (C). In toxin chains D–I, densities are largely complete except for minor gaps: G13 (D); R94–R95 (G); Q91–G93 (H); and G92–R95 (I). **(C)** The cartoon representation showing the conserved metal ion binding residues in the peptidase domain (shown in stick representation) bound to the Zn^2+^ ion. (**D)** MSA analysis of VapB52 with other IRRE family peptidases highlighting conserved Zn²⁺-binding motif (HEXXH) marked with asterisks. **(E)** Structural superposition of VapC52^D6A^ dimer with the VapC52 dimer bound to VapB52^Δ23^. The major conformational differences, due to domain motion, observed upon superposition are shown in a broken circle**. (F)** Interaction network at the VapB52^Δ23^–VapC52 interface I and interface II (Fig. 2A) stabilizing the TA complex through hydrogen bonds, salt bridges and non-bonded interactions. **(G)** Electrostatic potential (APBS) map of individual VapB52^Δ23^ and VapC52 showing surface charge distributionand asymmetric binding of VapB52^Δ23^ to only one active site (dotted circle) of VapC52.

**Supplementary Figure 4.** Construction of *vapC52* and *vapBC52* mutant strains of *Mtb.* ***(*A, B)** The schematic representation of *vapBC52* locus in wild type and deletion strains in which *vapC52* (**A**) and *vapBC52* (**B**) have been replaced with hygromycin resistance cassette using temperature-sensitive mycobacteriophages. **(C, D)** The construction of Δ*vapC52* and Δ*vapBC52* strains was confirmed by Southern blot using locus specific probes. **(E, F)** Thecolony morphology (lower) and biofilm formation (upper) of *ΔvapC52* (**E**) *ΔvapBC52* (**F**) and wild type strains was comparable. The data shown in (**E**) and (**F**) panels is representative of two independent experiments*.*

**Supplementary Figure 5.** VapC52 is dispensable for copper tolerance and intracellular growth of *Mtb* in macrophages and guinea pigs. **(A)** Intracellular survival in of various strains was determined in THP1 macrophages as described in methods. Data represent mean ± SD of log_10_ CFU obtained from two independent experiments performed in duplicate or triplicate. **(B)** Growth of wild type or Δ*vapC52 Mtb* strain on 7H11 medium in the absence or presence of copper was determined by spotting assays. **(C)** 6-8 week only female guinea pigs were infected with either parental or Δ*vapC52* strain of *Mtb* to establish ~100 bacilli in the lungs. The extent of disease progression in infected animals was determined at 4- and 8-weeks post-infection. **(D, E)** The data shown in this panel is mean ± SD of log_10_CFU obtained from lungs (**D**) and spleens (**E**) of guinea pigs at either 4 weeks or 8 weeks post-infection. **(F)** Representative gross pathology images of lungs from guinea pigs infected with wild type and *ΔvapC52* strain at 4 and 8 weeks post-infection. These images are representative of 5–6 guinea pigs per group.

**Supplementary Table 1**. Data collection, processing and refinement statistics

| **Protein** | **VapC52** | **VapB^Δ23^C52** |
| --- | --- | --- |
| PDB ID | 9VZZ | 9VZY |
| Space group | I 2 3 | C 2 2 2 |
| **Cell dimensions** |  |  |
| a, b, c (Å)  α, β, γ ( °) | 184.98, 184.98, 184.98  90, 90, 90 | 122.08, 202.32, 222.99  90, 90, 90 |
| Resolution (Å) | 39.44-2.6 (2.693-2.6) | 39.9-3.2 (3.314-3.2) |
| R_merge_ | 0.02178 (0.1722) | 0.06729 (0.63) |
| I/σI | 21.68 (4.36) | 6.85 (1.09) |
| Completeness (%) | 99.93 (99.94) | 99.78 (99.76) |
| Multiplicity | 2.0 (2.0) | 2.0 (2.0) |
| CC1/2 | 1 (0.918) | 0.997 (0.527) |
| **Refinement** |  |  |
| Resolution(Å) | 2.6 | 3.2 |
| Reflections | 32400 3203) | 45790 (4517) |
| R-work | 0.1719 (0.2080) | 0.2549 (0.4260) |
| R-free | 0.2293 (0.2754) | 0.2809 (0.4272) |
| **No. of Atom** |  |  |
| Protein | 4368 | 15544 |
| ligands | 46 | 5 |
| water | 272 | 92 |
| **B-factors (Å^2^ )** |  |  |
| Protein | 68.82 | 107.41 |
| ligands | 93.23 | 94.07 |
| water | 61.09 | 96.56 |
| **R.m.s deviations** |  |  |
| Bond lengths (Å) | 0.008 | 0.002 |
| Bond angles(°) | 1.34 | 0.53 |
| **Ramachandran plot statistics** |  |  |
| Most favoured (%) | 96.08 | 97.39 |
| Allowed regions (%) | 3.92 | 2.61 |

**Supplementary Table 2**. Structural homologs of VapC52 and VapB52 from PDBeFold server.

| **VapC52** | **S.No** | **Structural homologue** | **Organism** | **PDB ID** | **Z score** | **RMSD** | **Aligned Residues** | **Sequence identity (%)** |
| --- | --- | --- | --- | --- | --- | --- | --- | --- |
|  | 1 | PAE2754 | *Pyrobaculum aerophilumum* | 1V8P | 7.9 | 2.38 | 113 | 10 |
|  | 2 | VapC3 | *Mtb* | 3H87 | 7.7 | 2.11 | 109 | 13 |
|  | 3 | VapC43 | *Mtb* | 7VWO | 7.6 | 2.4 | 114 | 20 |
|  | 4 | SSO1118 | *Saccharolobus solfataricus* | 2MDT | 7.6 | 2.02 | 96 | 11 |
|  | 5 | VapC20 | *Mtb* | 5WZF | 7.4 | 2.4 | 103 | 13 |
|  | 6 | VapC11 | *Mtb* | 6A7V | 7 | 2.52 | 100 | 19 |
|  | 7 | VapC30 | *Mtb* | 4XGQ | 6.6 | 2.5 | 108 | 16 |
|  | 8 | VapC26 | *Mtb* | 5X3T | 6 | 2.29 | 107 | 18 |
| **VapB52 ^Δ23,NTD^** | 1 | C.CSP231I | *Citrobacter sp.* RFL231 | 4JCX:A | 8.6 | 0.96 | 63 | 19 |
|  | 2 | BswR | *Pseudomonas aeruginosa* PAO1 | 4O8B | 7.6 | 1.2 | 63 | 17 |
|  | 3 | HTH of DdrO | *Deinococcus deserti* | 6RNZ | 7.5 | 1.31 | 63 | 19 |
|  | 4 | DNA binding domain of SINR | *Bacillus subtilis* | 3QQ6 | 7.3 | 1.41 | 62 | 19 |
| **VapB52^CTD^** | 1 | Peroxidase Inhibitor SPIN | *Staphylococcus aureus* | 7Z53 | 4.1 | 2.75 | 42 | 10 |
|  | 2 | AvrptoB | *Pseudomonas syringae* | 3HGK | 3.8 | 2.04 | 40 | 10 |
|  | 3 | Aureocin A53 | *Staphylococcus aureus* | 8AVS | 3.3 | 3.04 | 33 | 12 |
|  | 4 | FKBP-rapamycin binding domain (FRB) | *Homo sapiens* | 6M4W | 3.2 | 2.55 | 38 | 8 |
| **VapB52 ^MD^** | 1 | CapP | *Thauera sp. K11* | 7T5T | 8.1 | 2.46 | 148 | 22 |
|  | 2 | IRRE | *Deinococcus deserti* | 3DTI | 7.8 | 1.83 | 117 | 25 |
|  | 3 | PrpR | *Mtb* | 6D2S | 7.1 | 2.3 | 127 | 17 |
|  | 4 | ArdC | *Escherichia coli* | 6SNA | 6.4 | 2.39 | 76 | 14 |
|  |  |  |  |  |  |  |  |  |

**Supplementary Table 3. Buried surface area comparison of VapC(A) toxins and VapBC complexes (B) using PDBePISA server.**

**A**

| S. No | Protein | Organism | oligomeric | Buried | ΔG^int^,  kcal/mol | ΔG^diss^,  kcal/mol |
| --- | --- | --- | --- | --- | --- | --- |
|  |  |  |  | area, sq. Å |  |  |
| 1 | VapC52**^D6A^** | *Mtb* | dimer | 1540 | -9.2 | 1.5 |
| 2 | PAE2754 | *Pyrobaculum*  *aerophilumum* | dimer | 2880 | -25.6 | 11 |
| 3 | VapC20 | *Mtb* | dimer | 2370 | -11 | 6.8 |
| 4 | VapC11 | *Mtb* | dimer | 2420 | -8.4 | 7.2 |

**B**

| S. No | Protein | Organism | Binding stoichiometry (T:AT) | Buried | ΔG^int^,   kcal/mol | ΔG^diss^,  kcal/mol |
| --- | --- | --- | --- | --- | --- | --- |
|  |  |  |  | area, sq. Å |  |  |
| 1 | VapB^Δ23^C52 | *Mtb* | 2:1 | 4800 | -13.9 | 0.7 |
| 2 | VapBC11 | *Mtb* | 1:1 | 2800 | -8.3 | 11 |
| 3 | VapBC15 | *Mtb* | 1:1 | 2990 | -29.2 | 16.9 |
| 4 | VapBC30 | *Mtb* | 1:1 | 1650 | -10.3 | 7.9 |

**Supplementary Table 4. The Log_2_ protection ratio and Log_2_ fold change in tRNAs levels upon VapC52 overexpression in *Mtb*.**

| **S. No** | **tRNA** | **Log_2_**  **protection ratio** | **Log_2_**  **fold change** |  | **S. No** | **tRNA** | **Log_2_**  **protection ratio** | **Log_2_**  **fold change** |
| --- | --- | --- | --- | --- | --- | --- | --- | --- |
|  |  |  |  |  |  |  |  |  |
| 1 | *glyU* | -3.797774 | -1.42405 |  | 15 | *valV* | -1.07491 | -0.21767 |
| 2 | *tyrT* | -1.326665 | -0.338962 |  | 16 | *asnT* | -4.40058 | -1.21498 |
| 3 | *thrT* | -0.786009 | -0.235771 |  | 17 | *argW* | -2.36399 | -1.79176 |
| 4 | *metT* | -0.514892 | 0.141527 |  | 18 | *hisT* | -3.77186 | -3.19851 |
| 5 | *trpT* | -1.381986 | 1.81566 |  | 19 | *valT* | -3.09627 | -3.37225 |
| 6 | *lysT* | -1.741703 | -1.1486 |  | 20 | *glyT* | -1.5975 | -1.93383 |
| 7 | *pheU* | -0.25922 | 0.210159 |  | 21 | *cysU* | -0.97671 | -0.43726 |
| 8 | *argT* | -1.669557 | -1.93252 |  | 22 | *valU* | -2.57144 | -2.32746 |
| 9 | *glnT* | 0.374937 | 0.0348552 |  | 23 | *gluU* | -2.7417 | -1.28648 |
| 10 | *argV* | 0.931821 | 1.87716 |  | 24 | *glnU* | -2.20087 | -0.02428 |
| 11 | *leuW* | -2.105279 | -1.66134 |  | 25 | *alaU* | -2.6869 | -2.72991 |
| 12 | *leuu* | -1.783391 | -2.19859 |  | 26 | *metU* | -2.4815 | -1.7189 |
| 13 | *SerT* | -1.43895 | -0.51527 |  | 27 | *serV* | -3.99163 | -2.81664 |
| 14 | *SerU* | -3.10528 | -2.57268 |  | 28 | *serX* | -3.17207 | -2.63883 |
